# Supplementary material for: Enhancement of conformational B-cell epitope prediction using CluSMOTE
Source: PeerJ Comput Sci. 2020 Jun 1;6:e275. doi: 10.7717/peerj-cs.275 (PMC7924438; doi:10.7717/peerj-cs.275)
Supplement: Supplemental Information 1 [file peerj-cs-06-275-s001.docx]

**Suplementary Tables**

Table 1. List of Antigen Chain for The Training Set

| No | PDBID | Ag Chain | No | PDBID | Ag Chain | No | PDBID | Ag Chain |
| --- | --- | --- | --- | --- | --- | --- | --- | --- |
| 1 | 1A2Y | C | 21 | 1HYS | A,B | 41 | 1QLE | B |
| 2 | 1ADQ | A | 22 | 1I9R | A | 42 | 1RVF | 1,2,3,4 |
| 3 | 1AFV | A | 23 | 1IAI | H,L | 43 | 1SY6 | A |
| 4 | 1AHW | C | 24 | 1IGC | A | 44 | 1TPX | A |
| 5 | 1AR1 | A, B | 25 | 1IQD | C | 45 | 1V7M | V |
| 6 | 1BGX | T | 26 | 1JHL | A | 46 | 1WEJ | F |
| 7 | 1BJ1 | V | 27 | 1JRH | I | 47 | 2JEL | P |
| 8 | 1BQL | Y | 28 | 1K4D | C | 48 | 2VXQ | A |
| 9 | 1CIC | C,D | 29 | 1KB5 | A,B | 49 | 2W9E | A |
| 10 | 1DVF | A,B | 30 | 1LK3 | A | 50 | 3C09 | A |
| 11 | 1E6J | P | 31 | 1MHP | A | 51 | 3CSY | O,P |
| 12 | 1EGJ | A | 32 | 1N8Z | C | 52 | 3EO1 | C,F |
| 13 | 1EO8 | A,B | 33 | 1NCA | N | 53 | 3EOA | A |
| 14 | 1EZV | E | 34 | 1NFD | A,B | 54 | 3FMG | A |
| 15 | 1FE8 | A | 35 | 1OAZ | A | 55 | 3G6D | A |
| 16 | 1FJ1 | F | 36 | 1ORQ | C | 56 | 3G6J | A,B |
| 17 | 1FNS | A | 37 | 1OTS | A | 57 | 3GBN | A,B |
| 18 | 1FSK | A | 38 | 1PG7 | H,L | 58 | 3GRW | A |
| 19 | 1G9M | G | 39 | 1PKQ | E | 59 | 3H42 | A |
| 20 | 1H0D | C | 40 | 1QFW | A | 60 | 1FBI | X |

Table 2a List of Antigen Chain part of Kringelum as independent test set

| No | PDBID | Ag Chain | No | PDBID | Ag Chain | No | PDBID | Ag Chain |
| --- | --- | --- | --- | --- | --- | --- | --- | --- |
| 1 | 1YNT | F | 14 | 3GI8 | C | 27 | 2ZCH | P |
| 2 | 2NYY | A | 15 | 3GI9 | C | 28 | 2ZCK | P |
| 3 | 2NZ9 | A | 16 | 3NCY | C,D | 29 | 2ZCL | P |
| 4 | 3I50 | E | 17 | 2DD8 | S | 30 | 3BN9 | B |
| 5 | 1Z3G | A | 18 | 2GHW | A | 31 | 3L95 | Y |
| 6 | 2R56 | A,B | 19 | 3BGF | S | 32 | 3MXW | A |
| 7 | 3BSZ | E,F | 20 | 2XQY | A | 33 | 2R4R | A |
| 8 | 2I9L | I | 21 | 2NR6 | A | 34 | 2R4S | A |
| 9 | 3G04 | C | 22 | 3LIZ | A | 35 | 3KJ6 | A |
| 10 | 3KJ4 | A | 23 | 2ZJS | Y | 36 | 2OZ4 | A |
| 11 | 3O0R | B,C | 24 | 2UZI | R | 37 | 2FD6 | U |
| 12 | 2Q8A | A | 25 | 2VH5 | R | 38 | 3PGF | A |
| 13 | 2Q8B | A | 26 | 3R1G | B | 39 | 2J88 | A |

Table 2b. List of Antigen Chain part of Chou as independent test set

| No | PDBID | Ag Chain | No | PDBID | Ag Chain | No | PDBID | Ag Chain |
| --- | --- | --- | --- | --- | --- | --- | --- | --- |
| 1 | 3JCX | A | 31 | 5IES | C | 61 | 5L0Q | A |
| 2 | 5B3J | A,D | 32 | 5IF0 | G | 62 | 5L6Y | C |
| 3 | 5B8C | C | 33 | 5IGX | G | 63 | 5M94 | A |
| 4 | 5FUU | C,F | 34 | 5J3H | E | 64 | 5MVZ | U |
| 5 | 5FV1 | V | 35 | 5J56 | A | 65 | 5SV3 | B |
| 6 | 5FV2 | X | 36 | 5J57 | A | 66 | 5SX4 | N |
| 7 | 5FYL | G | 37 | 5JQ6 | A | 67 | 5SX5 | N |
| 8 | 5FYL | B | 38 | 5JS9 | C | 68 | 5T33 | G |
| 9 | 5GGR | Z | 39 | 5JS9 | D | 69 | 5T3S | G |
| 10 | 5GGS | Z | 40 | 5JSA | D | 70 | 5T3Z | G |
| 11 | 5GGV | Y | 41 | 5JW3 | A,B | 71 | 5TE4 | G |
| 12 | 5GJS | A | 42 | 5JW4 | A | 72 | 5TE6 | G |
| 13 | 5GJS | B | 43 | 5JXE | B | 73 | 5TE7 | G |
| 14 | 5GJT | A,B | 44 | 5JYL | A | 74 | 5TH9 | A |
| 15 | 5GRJ | A | 45 | 5JYM | A | 75 | 5THR | C |
| 16 | 5GS0 | A | 46 | 5JZ7 | B | 76 | 5THR | D |
| 17 | 5GZN | A | 47 | 5K59 | A | 77 | 5TOJ | A |
| 18 | 5H37 | A | 48 | 5K9K | I | 78 | 5TOK | B |
| 19 | 5HDZ | A | 49 | 5K9O | I,F | 79 | 5TPN | A |
| 20 | 5HHX | A | 50 | 5KAN | C,D | 80 | 5TPW | A |
| 21 | 5HI3 | B | 51 | 5KAQ | B | 81 | 5TQ0 | A |
| 22 | 5HI4 | A | 52 | 5KEL | B | 82 | 5TQ2 | A |
| 23 | 5HI5 | A | 53 | 5KEL | A | 83 | 5TQQ | A |
| 24 | 5HJ3 | D | 54 | 5KEN | E | 84 | 5TR1 | A |
| 25 | 5I6X | A | 55 | 5KEN | B | 85 | 5TZ2 | C |
| 26 | 5I6Z | A | 56 | 5KJR | G | 86 | 5TZT | C |
| 27 | 5I74 | A | 57 | 5KOV | A | 87 | 5TZU | C |
| 28 | 5I8H | C | 58 | 5KTE | A | 88 | 5UGY | A |
| 29 | 5I8H | B | 59 | 5KW9 | A | 89 | 5UQY | E |
| 30 | 5I9Q | G | 60 | 5KZC | A | 90 | 5WT9 | G |

Table 3. Index component of AAIndex in aaindex.txt

| Header | A/L R/K N/M D/F C/P Q/S E/T G/W H/Y I/V |
| --- | --- |
| ANDN920101 | 4.35 4.38 4.75 4.76 4.65 4.37 4.29 3.97 4.63 3.95 4.17 4.36 4.52 4.66 4.44 4.5 4.35 4.7 4.6 3.95 |
| ARGP820101 | 0.61 0.6 0.06 0.46 1.07 0.0 0.47 0.07 0.61 2.22 1.53 1.15 1.18 2.02 1.95 0.05 0.05 2.65 1.88 1.32 |
| ARGP820102 | 1.18 0.2 0.23 0.05 1.89 0.72 0.11 0.49 0.31 1.45  3.23 0.06 2.67 1.96 0.76 0.97 0.84 0.77 0.39 1.08 |
| ARGP820103 | 1.56 0.45 0.27 0.14 1.23 0.51 0.23 0.62 0.29 1.67  2.93 0.15 2.96 2.03 0.76 0.81 0.91 1.08 0.68 1.14 |
| BEGF750101 | 1.0 0.52 0.35 0.44 0.06 0.44 0.73 0.35 0.6 0.73  1.0 0.6 1.0 0.6 0.06 0.35 0.44 0.73 0.44 0.82 |
| BEGF750102 | 0.77 0.72 0.55 0.65 0.65 0.72 0.55 0.65 0.83 0.98  0.83 0.55 0.98 0.98 0.55 0.55 0.83 0.77 0.83 0.98 |
| BEGF750103 | 0.37 0.84 0.97 0.97 0.84 0.64 0.53 0.97 0.75 0.37  0.53 0.75 0.64 0.53 0.97 0.84 0.75 0.97 0.84 0.37 |
| BHAR880101 | 0.357 0.529 0.463 0.511 0.346 0.493 0.497 0.544 0.323 0.462 0.365 0.466 0.295 0.314 0.509 0.507 0.444 0.305 0.42 0.386 |
| BIGC670101 | 52.6 109.1 75.7 68.4 68.3 89.7 84.7 36.3 91.9 102.0 102.0 105.1 97.7 113.9 73.6 54.9 71.2 135.4 116.2 85.1 |
| BIOV880101 | 16.0 -70.0 -74.0 -78.0 168.0 -73.0 -106.0 -13.0 50.0 151.0  145.0 -141.0 124.0 189.0 -20.0 -70.0 -38.0 145.0 53.0 123.0 |
| BIOV880102 | 44.0 -68.0 -72.0 -91.0 90.0 -117.0 -139.0 -8.0 47.0 100.0  108.0 -188.0 121.0 148.0 -36.0 -60.0 -54.0 163.0 22.0 117.0 |
| BROC820101 | 7.3 -3.6 -5.7 -2.9 -9.2 -0.3 -7.1 -1.2 -2.1 6.6  20.0 -3.7 5.6 19.2 5.1 -4.1 0.8 16.3 5.9 3.5 |
| BROC820102 | 3.9 3.2 -2.8 -2.8 -14.3 1.8 -7.5 -2.3 2.0 11.0  15.0 -2.5 4.1 14.7 5.6 -3.5 1.1 17.8 3.8 2.1 |
| BULH740101 | -0.2 -0.12 0.08 -0.2 -0.45 0.16 -0.3 0.0 -0.12 -2.26  -2.46 -0.35 -1.47 -2.33 -0.98 -0.39 -0.52 -2.01 -2.24 -1.56 |
| BULH740102 | 0.691 0.728 0.596 0.558 0.624 0.649 0.632 0.592 0.646 0.809  0.842 0.767 0.709 0.756 0.73 0.594 0.655 0.743 0.743 0.777 |
| BUNA790101 | 8.249 8.274 8.747 8.41 8.312 8.411 8.368 8.391 8.415 8.195  8.423 8.408 8.418 8.228 0.0 8.38 8.236 8.094 8.183 8.436 |
| BUNA790102 | 4.349 4.396 4.755 4.765 4.686 4.373 4.295 3.972 4.63 4.224  4.385 4.358 4.513 4.663 4.471 4.498 4.346 4.702 4.604 4.184 |
| BUNA790103 | 6.5 6.9 7.5 7.0 7.7 6.0 7.0 5.6 8.0 7.0  6.5 6.5 0.0 9.4 0.0 6.5 6.9 0.0 6.8 7.0 |
| BURA740101 | 0.486 0.262 0.193 0.288 0.2 0.418 0.538 0.12 0.4 0.37  0.42 0.402 0.417 0.318 0.208 0.2 0.272 0.462 0.161 0.379 |
| BURA740102 | 0.288 0.362 0.229 0.271 0.533 0.327 0.262 0.312 0.2 0.411 0.4 0.265 0.375 0.318 0.34 0.354 0.388 0.231 0.429 0.495 |
| CHAM810101 | 0.52 0.68 0.76 0.76 0.62 0.68 0.68 0.0 0.7 1.02  0.98 0.68 0.78 0.7 0.36 0.53 0.5 0.7 0.7 0.76 |
| CHAM820101 | 0.046 0.291 0.134 0.105 0.128 0.18 0.151 0.0 0.23 0.186  0.186 0.219 0.221 0.29 0.131 0.062 0.108 0.409 0.298 0.14 |
| CHAM820102 | 0.71 1.06 1.37 1.21 1.19 0.87 0.84 1.52 1.07 0.66  0.69 0.99 0.59 0.71 1.61 1.34 1.08 0.76 1.07 0.63 |
| CHAM830101 | -0.118 0.124 0.289 0.048 0.083 -0.105 -0.245 0.104 0.138 0.23  -0.052 0.032 -0.258 0.015 0.0 0.225 0.166 0.158 0.094 0.513 |
| CHAM830102 | 0.0 1.0 1.0 1.0 1.0 1.0 1.0 0.0 1.0 2.0  1.0 1.0 1.0 1.0 0.0 1.0 2.0 1.0 1.0 2.0 |
| CHAM830103 | 0.0 1.0 1.0 1.0 0.0 1.0 1.0 0.0 1.0 1.0  2.0 1.0 1.0 1.0 0.0 0.0 0.0 1.0 1.0 0.0 |
| CHAM830104 | 0.0 1.0 0.0 0.0 0.0 1.0 1.0 0.0 1.0 0.0  0.0 1.0 1.0 1.0 0.0 0.0 0.0 1.5 1.0 0.0 |
| CHAM830105 | 0.0 5.0 2.0 2.0 1.0 3.0 3.0 0.0 3.0 2.0  2.0 4.0 3.0 4.0 0.0 1.0 1.0 5.0 5.0 1.0 |
| CHAM830106 | 0.0 0.0 1.0 1.0 0.0 0.0 1.0 1.0 0.0 0.0  0.0 0.0 0.0 0.0 0.0 0.0 0.0 0.0 0.0 0.0 |
| CHAM830107 | 0.0 1.0 1.0 0.0 1.0 1.0 0.0 0.0 1.0 0.0  0.0 1.0 1.0 1.0 0.0 0.0 0.0 1.0 1.0 0.0 |
| CHAM830108 | 91.5 202.0 135.2 124.5 117.7 161.1 155.1 66.4 167.3 168.8  167.9 171.3 170.8 203.4 129.3 99.1 122.1 237.6 203.6 141.7 |
| CHOC750101 | 115.0 225.0 160.0 150.0 135.0 180.0 190.0 75.0 195.0 175.0  170.0 200.0 185.0 210.0 145.0 115.0 140.0 255.0 230.0 155.0 |
| CHOC760101 | 25.0 90.0 63.0 50.0 19.0 71.0 49.0 23.0 43.0 18.0  23.0 97.0 31.0 24.0 50.0 44.0 47.0 32.0 60.0 18.0 |
| CHOC760102 | 0.38 0.01 0.12 0.15 0.45 0.07 0.18 0.36 0.17 0.6  0.45 0.03 0.4 0.5 0.18 0.22 0.23 0.27 0.15 0.54 |
| CHOC760103 | 0.2 0.0 0.03 0.04 0.22 0.01 0.03 0.18 0.02 0.19  0.16 0.0 0.11 0.14 0.04 0.08 0.08 0.04 0.03 0.18 |
| CHOC760104 | 0.66 0.95 1.56 1.46 1.19 0.98 0.74 1.56 0.95 0.47  0.59 1.01 0.6 0.6 1.52 1.43 0.96 0.96 1.14 0.5 |
| CHOP780101 | 1.42 0.98 0.67 1.01 0.7 1.11 1.51 0.57 1.0 1.08  1.21 1.16 1.45 1.13 0.57 0.77 0.83 1.08 0.69 1.06 |
| CHOP780201 | 0.83 0.93 0.89 0.54 1.19 1.1 0.37 0.75 0.87 1.6  1.3 0.74 1.05 1.38 0.55 0.75 1.19 1.37 1.47 1.7 |
| CHOP780202 | 0.74 1.01 1.46 1.52 0.96 0.96 0.95 1.56 0.95 0.47  0.5 1.19 0.6 0.66 1.56 1.43 0.98 0.6 1.14 0.59 |
| CHOP780203 | 1.29 0.44 0.81 2.02 0.66 1.22 2.44 0.76 0.73 0.67  0.58 0.66 0.71 0.61 2.01 0.74 1.08 1.47 0.68 0.61 |
| CHOP780204 | 1.2 1.25 0.59 0.61 1.11 1.22 1.24 0.42 1.77 0.98  1.13 1.83 1.57 1.1 0.0 0.96 0.75 0.4 0.73 1.25 |
| CHOP780205 | 0.7 0.34 1.42 0.98 0.65 0.75 1.04 1.41 1.22 0.78  0.85 1.01 0.83 0.93 1.1 1.55 1.09 0.62 0.99 0.75 |
| CHOP780206 | 0.52 1.24 1.64 1.06 0.94 0.7 0.59 1.64 1.86 0.87  0.84 1.49 0.52 1.04 1.58 0.93 0.86 0.16 0.96 0.32 |
| CHOP780207 | 0.86 0.9 0.66 0.38 0.87 1.65 0.35 0.63 0.54 1.94  1.3 1.0 1.43 1.5 0.66 0.63 1.17 1.49 1.07 1.69 |
| CHOP780208 | 0.75 0.9 1.21 0.85 1.11 0.65 0.55 0.74 0.9 1.35  1.27 0.74 0.95 1.5 0.4 0.79 0.75 1.19 1.96 1.79 |
| CHOP780209 | 0.67 0.89 1.86 1.39 1.34 1.09 0.92 1.46 0.78 0.59  0.46 1.09 0.52 0.3 1.58 1.41 1.09 0.48 1.23 0.42 |
| CHOP780210 | 0.74 1.05 1.13 1.32 0.53 0.77 0.85 1.68 0.96 0.53  0.59 0.82 0.85 0.44 1.69 1.49 1.16 1.59 1.01 0.59 |
| CHOP780211 | 0.06 0.07 0.161 0.147 0.149 0.074 0.056 0.102 0.14 0.043  0.061 0.055 0.068 0.059 0.102 0.12 0.086 0.077 0.082 0.062 |
| CHOP780212 | 0.076 0.106 0.083 0.11 0.053 0.098 0.06 0.085 0.047 0.034  0.025 0.115 0.082 0.041 0.301 0.139 0.108 0.013 0.065 0.048 |
| CHOP780213 | 0.035 0.099 0.191 0.179 0.117 0.037 0.077 0.19 0.093 0.013  0.036 0.072 0.014 0.065 0.034 0.125 0.065 0.064 0.114 0.028 |
| CHOP780214 | 0.058 0.085 0.091 0.081 0.128 0.098 0.064 0.152 0.054 0.056  0.07 0.095 0.055 0.065 0.068 0.106 0.079 0.167 0.125 0.053 |
| CHOP780215 | 0.64 1.05 1.56 1.61 0.92 0.84 0.8 1.63 0.77 0.29  0.36 1.13 0.51 0.62 2.04 1.52 0.98 0.48 1.08 0.43 |
| CHOP780216 | -0.45 -0.24 -0.2 -1.52 0.79 -0.99 -0.8 -1.0 1.07 0.76  1.29 -0.36 1.37 1.48 -0.12 -0.98 -0.7 1.38 1.49 1.26 |
| CIDH920101 | -0.08 -0.09 -0.7 -0.71 0.76 -0.4 -1.31 -0.84 0.43 1.39  1.24 -0.09 1.27 1.53 -0.01 -0.93 -0.59 2.25 1.53 1.09 |
| CIDH920102 | 0.36 -0.52 -0.9 -1.09 0.7 -1.05 -0.83 -0.82 0.16 2.17  1.18 -0.56 1.21 1.01 -0.06 -0.6 -1.2 1.31 1.05 1.21 |
| CIDH920103 | 0.17 -0.7 -0.9 -1.05 1.24 -1.2 -1.19 -0.57 -0.25 2.06  0.96 -0.62 0.6 1.29 -0.21 -0.83 -0.62 1.51 0.66 1.21 |
| CIDH920104 | 0.02 -0.42 -0.77 -1.04 0.77 -1.1 -1.14 -0.8 0.26 1.81  1.14 -0.41 1.0 1.35 -0.09 -0.97 -0.77 1.71 1.11 1.13 |
| CIDH920105 | 0.75 0.7 0.61 0.6 0.61 0.67 0.66 0.64 0.67 0.9  0.9 0.82 0.75 0.77 0.76 0.68 0.7 0.74 0.71 0.86 |
| COHE430101 | 1.33 0.79 0.72 0.97 0.93 1.42 1.66 0.58 1.49 0.99  1.29 1.03 1.4 1.15 0.49 0.83 0.94 1.33 0.49 0.96 |
| CRAJ730101 | 1.0 0.74 0.75 0.89 0.99 0.87 0.37 0.56 0.36 1.75  1.53 1.18 1.4 1.26 0.36 0.65 1.15 0.84 1.41 1.61 |
| CRAJ730102 | 0.6 0.79 1.42 1.24 1.29 0.92 0.64 1.38 0.95 0.67  0.7 1.1 0.67 1.05 1.47 1.26 1.05 1.23 1.35 0.48 |
| CRAJ730103 | 2.5 7.5 5.0 2.5 3.0 6.0 5.0 0.5 6.0 5.5  5.5 7.0 6.0 6.5 5.5 3.0 5.0 7.0 7.0 5.0 |
| DAWD720101 | 8.6 4.9 4.3 5.5 2.9 3.9 6.0 8.4 2.0 4.5  7.4 6.6 1.7 3.6 5.2 7.0 6.1 1.3 3.4 6.6 |
| DAYM780101 | 100.0 65.0 134.0 106.0 20.0 93.0 102.0 49.0 66.0 96.0  40.0 56.0 94.0 41.0 56.0 120.0 97.0 18.0 41.0 74.0 |
| DAYM780201 | 1.56 0.59 0.51 0.23 1.8 0.39 0.19 1.03 1.0 1.27  1.38 0.15 1.93 1.42 0.27 0.96 1.11 0.91 1.1 1.58 |
| DESM900101 | 1.26 0.38 0.59 0.27 1.6 0.39 0.23 1.08 1.0 1.44  1.36 0.33 1.52 1.46 0.54 0.98 1.01 1.06 0.89 1.33 |
| DESM900102 | 0.25 -1.76 -0.64 -0.72 0.04 -0.69 -0.62 0.16 -0.4 0.73  0.53 -1.1 0.26 0.61 -0.07 -0.26 -0.18 0.37 0.02 0.54 |
| EISD840101 | 0.67 -2.1 -0.6 -1.2 0.38 -0.22 -0.76 0.0 0.64 1.9  1.9 -0.57 2.4 2.3 1.2 0.01 0.52 2.6 1.6 1.5 |
| EISD860101 | 0.0 10.0 1.3 1.9 0.17 1.9 3.0 0.0 0.99 1.2  1.0 5.7 1.9 1.1 0.18 0.73 1.5 1.6 1.8 0.48 |
| EISD860102 | 0.0 -0.96 -0.86 -0.98 0.76 -1.0 -0.89 0.0 -0.75 0.99  0.89 -0.99 0.94 0.92 0.22 -0.67 0.09 0.67 -0.93 0.84 |
| EISD860103 | 89.09 174.2 132.12 133.1 121.15 146.15 147.13 75.07 155.16 131.17  131.17 146.19 149.21 165.19 115.13 105.09 119.12 204.24 181.19 117.15 |
| FASG760101 | 297.0 238.0 236.0 270.0 178.0 185.0 249.0 290.0 277.0 284.0  337.0 224.0 283.0 284.0 222.0 228.0 253.0 282.0 344.0 293.0 |
| FASG760102 | 1.8 12.5 -5.6 5.05 -16.5 6.3 12.0 0.0 -38.5 12.4  -11.0 14.6 -10.0 -34.5 -86.2 -7.5 -28.0 -33.7 -10.0 5.63 |
| FASG760103 | 9.69 8.99 8.8 9.6 8.35 9.13 9.67 9.78 9.17 9.68  9.6 9.18 9.21 9.18 10.64 9.21 9.1 9.44 9.11 9.62 |
| FASG760104 | 2.34 1.82 2.02 1.88 1.92 2.17 2.1 2.35 1.82 2.36  2.36 2.16 2.28 2.16 1.95 2.19 2.09 2.43 2.2 2.32 |
| FASG760105 | 0.31 -1.01 -0.6 -0.77 1.54 -0.22 -0.64 0.0 0.13 1.8  1.7 -0.99 1.23 1.79 0.72 -0.04 0.26 2.25 0.96 1.22 |
| FAUJ830101 | 1.28 2.34 1.6 1.6 1.77 1.56 1.56 0.0 2.99 4.19  2.59 1.89 2.35 2.94 2.67 1.31 3.03 3.21 2.94 3.67 |
| FAUJ880101 | 0.53 0.69 0.58 0.59 0.66 0.71 0.72 0.0 0.64 0.96  0.92 0.78 0.77 0.71 0.0 0.55 0.63 0.84 0.71 0.89 |
| FAUJ880102 | 1.0 6.13 2.95 2.78 2.43 3.95 3.78 0.0 4.66 4.0  4.0 4.77 4.43 5.89 2.72 1.6 2.6 8.08 6.47 3.0 |
| FAUJ880103 | 2.87 7.82 4.58 4.74 4.47 6.11 5.97 2.06 5.23 4.92  4.92 6.89 6.36 4.62 4.11 3.97 4.11 7.68 4.73 4.11 |
| FAUJ880104 | 1.52 1.52 1.52 1.52 1.52 1.52 1.52 1.0 1.52 1.9  1.52 1.52 1.52 1.52 1.52 1.52 1.73 1.52 1.52 1.9 |
| FAUJ880105 | 2.04 6.24 4.37 3.78 3.41 3.53 3.31 1.0 5.66 3.49  4.45 4.87 4.8 6.02 4.31 2.7 3.17 5.9 6.72 3.17 |
| FAUJ880106 | 7.3 11.1 8.0 9.2 14.4 10.6 11.4 0.0 10.2 16.1  10.1 10.9 10.4 13.9 17.8 13.1 16.7 13.2 13.9 17.2 |
| FAUJ880107 | -0.01 0.04 0.06 0.15 0.12 0.05 0.07 0.0 0.08 -0.01  -0.01 0.0 0.04 0.03 0.0 0.11 0.04 0.0 0.03 0.01 |
| FAUJ880108 | 0.0 4.0 2.0 1.0 0.0 2.0 1.0 0.0 1.0 0.0  0.0 2.0 0.0 0.0 0.0 1.0 1.0 1.0 1.0 0.0 |
| FAUJ880109 | 0.0 3.0 3.0 4.0 0.0 3.0 4.0 0.0 1.0 0.0  0.0 1.0 0.0 0.0 0.0 2.0 2.0 0.0 2.0 0.0 |
| FAUJ880110 | 0.0 1.0 0.0 0.0 0.0 0.0 0.0 0.0 1.0 0.0  0.0 1.0 0.0 0.0 0.0 0.0 0.0 0.0 0.0 0.0 |
| FAUJ880111 | 0.0 0.0 0.0 1.0 0.0 0.0 1.0 0.0 0.0 0.0  0.0 0.0 0.0 0.0 0.0 0.0 0.0 0.0 0.0 0.0 |
| FAUJ880112 | 4.76 4.3 3.64 5.69 3.67 4.54 5.48 3.77 2.84 4.81  4.79 4.27 4.25 4.31 0.0 3.83 3.87 4.75 4.3 4.86 |
| FAUJ880113 | 1.08 1.05 0.85 0.85 0.95 0.95 1.15 0.55 1.0 1.05  1.25 1.15 1.15 1.1 0.71 0.75 0.75 1.1 1.1 0.95 |
| FINA770101 | 1.0 0.7 1.7 3.2 1.0 1.0 1.7 1.0 1.0 0.6  1.0 0.7 1.0 1.0 1.0 1.7 1.7 1.0 1.0 0.6 |
| FINA910101 | 1.0 0.7 1.0 1.7 1.0 1.0 1.7 1.3 1.0 1.0  1.0 0.7 1.0 1.0 13.0 1.0 1.0 1.0 1.0 1.0 |
| FINA910102 | 1.2 1.7 1.2 0.7 1.0 1.0 0.7 0.8 1.2 0.8  1.0 1.7 1.0 1.0 1.0 1.5 1.0 1.0 1.0 0.8 |
| FINA910103 | 1.0 1.7 1.0 0.7 1.0 1.0 0.7 1.5 1.0 1.0  1.0 1.7 1.0 1.0 0.1 1.0 1.0 1.0 1.0 1.0 |
| FINA910104 | 0.28 0.1 0.25 0.21 0.28 0.35 0.33 0.17 0.21 0.82  1.0 0.09 0.74 2.18 0.39 0.12 0.21 5.7 1.26 0.6 |
| GARJ730101 | 1.29 1.0 0.81 1.1 0.79 1.07 1.49 0.63 1.33 1.05  1.31 1.33 1.54 1.13 0.63 0.78 0.77 1.18 0.71 0.81 |
| GEIM800101 | 1.13 1.09 1.06 0.94 1.32 0.93 1.2 0.83 1.09 1.05  1.13 1.08 1.23 1.01 0.82 1.01 1.17 1.32 0.88 1.13 |
| GEIM800102 | 1.55 0.2 1.2 1.55 1.44 1.13 1.67 0.59 1.21 1.27  1.25 1.2 1.37 0.4 0.21 1.01 0.55 1.86 1.08 0.64 |
| GEIM800103 | 1.19 1.0 0.94 1.07 0.95 1.32 1.64 0.6 1.03 1.12  1.18 1.27 1.49 1.02 0.68 0.81 0.85 1.18 0.77 0.74 |
| GEIM800104 | 0.84 1.04 0.66 0.59 1.27 1.02 0.57 0.94 0.81 1.29  1.1 0.86 0.88 1.15 0.8 1.05 1.2 1.15 1.39 1.56 |
| GEIM800105 | 0.86 1.15 0.6 0.66 0.91 1.11 0.37 0.86 1.07 1.17  1.28 1.01 1.15 1.34 0.61 0.91 1.14 1.13 1.37 1.31 |
| GEIM800106 | 0.91 0.99 0.72 0.74 1.12 0.9 0.41 0.91 1.01 1.29  1.23 0.86 0.96 1.26 0.65 0.93 1.05 1.15 1.21 1.58 |
| GEIM800107 | 0.91 1.0 1.64 1.4 0.93 0.94 0.97 1.51 0.9 0.65  0.59 0.82 0.58 0.72 1.66 1.23 1.04 0.67 0.92 0.6 |
| GEIM800108 | 0.8 0.96 1.1 1.6 0.0 1.6 0.4 2.0 0.96 0.85  0.8 0.94 0.39 1.2 2.1 1.3 0.6 0.0 1.8 0.8 |
| GEIM800109 | 1.1 0.93 1.57 1.41 1.05 0.81 1.4 1.3 0.85 0.67  0.52 0.94 0.69 0.6 1.77 1.13 0.88 0.62 0.41 0.58 |
| GEIM800111 | 0.93 1.01 1.36 1.22 0.92 0.83 1.05 1.45 0.96 0.58  0.59 0.91 0.6 0.71 1.67 1.25 1.08 0.68 0.98 0.62 |
| GOLD730101 | 0.75 0.75 0.69 0.0 1.0 0.59 0.0 0.0 0.0 2.95  2.4 1.5 1.3 2.65 2.6 0.0 0.45 3.0 2.85 1.7 |
| GOLD730102 | 88.3 181.2 125.1 110.8 112.4 148.7 140.5 60.0 152.6 168.5  168.5 175.6 162.2 189.0 122.2 88.7 118.2 227.0 193.0 141.4 |
| GRAR740101 | 0.0 0.65 1.33 1.38 2.75 0.89 0.92 0.74 0.58 0.0  0.0 0.33 0.0 0.0 0.39 1.42 0.71 0.13 0.2 0.0 |
| GRAR740102 | 8.1 10.5 11.6 13.0 5.5 10.5 12.3 9.0 10.4 5.2  4.9 11.3 5.7 5.2 8.0 9.2 8.6 5.4 6.2 5.9 |
| GRAR740103 | 31.0 124.0 56.0 54.0 55.0 85.0 83.0 3.0 96.0 111.0  111.0 119.0 105.0 132.0 32.5 32.0 61.0 170.0 136.0 84.0 |
| GUYH850101 | 0.1 1.91 0.48 0.78 -1.42 0.95 0.83 0.33 -0.5 -1.13  -1.18 1.4 -1.59 -2.12 0.73 0.52 0.07 -0.51 -0.21 -1.27 |
| HOPA770101 | 1.0 2.3 2.2 6.5 0.1 2.1 6.2 1.1 2.8 0.8  0.8 5.3 0.7 1.4 0.9 1.7 1.5 1.9 2.1 0.9 |
| HOPT810101 | -0.5 3.0 0.2 3.0 -1.0 0.2 3.0 0.0 -0.5 -1.8  -1.8 3.0 -1.3 -2.5 0.0 0.3 -0.4 -3.4 -2.3 -1.5 |
| HUTJ700101 | 29.22 26.37 38.3 37.09 50.7 44.02 41.84 23.71 59.64 45.0  48.03 57.1 69.32 48.52 36.13 32.4 35.2 56.92 51.73 40.35 |
| HUTJ700102 | 30.88 68.43 41.7 40.66 53.83 46.62 44.98 24.74 65.99 49.71  50.62 63.21 55.32 51.06 39.21 35.65 36.5 60.0 51.15 42.75 |
| HUTJ700103 | 154.33 341.01 207.9 194.91 219.79 235.51 223.16 127.9 242.54 233.21  232.3 300.46 202.65 204.74 179.93 174.06 205.8 237.01 229.15 207.6 |
| ISOY800101 | 1.53 1.17 0.6 1.0 0.89 1.27 1.63 0.44 1.03 1.07  1.32 1.26 1.66 1.22 0.25 0.65 0.86 1.05 0.7 0.93 |
| ISOY800102 | 0.86 0.98 0.74 0.69 1.39 0.89 0.66 0.7 1.06 1.31  1.01 0.77 1.06 1.16 1.16 1.09 1.24 1.17 1.28 1.4 |
| ISOY800103 | 0.78 1.06 1.56 1.5 0.6 0.78 0.97 1.73 0.83 0.4  0.57 1.01 0.3 0.67 1.55 1.19 1.09 0.74 1.14 0.44 |
| ISOY800104 | 1.09 0.97 1.14 0.77 0.5 0.83 0.92 1.25 0.67 0.66  0.44 1.25 0.45 0.5 2.96 1.21 1.33 0.62 0.94 0.56 |
| ISOY800105 | 0.35 0.75 2.12 2.16 0.5 0.73 0.65 2.4 1.19 0.12  0.58 0.83 0.22 0.89 0.43 1.24 0.85 0.62 1.44 0.43 |
| ISOY800106 | 1.09 1.07 0.88 1.24 1.04 1.09 1.14 0.27 1.07 0.97  1.3 1.2 0.55 0.8 1.78 1.2 0.99 1.03 0.69 0.77 |
| ISOY800107 | 1.34 2.78 0.92 1.77 1.44 0.79 2.54 0.95 0.0 0.52  1.05 0.79 0.0 0.43 0.37 0.87 1.14 1.79 0.73 0.0 |
| ISOY800108 | 0.47 0.52 2.16 1.15 0.41 0.95 0.64 3.03 0.89 0.62  0.53 0.98 0.68 0.61 0.63 1.03 0.39 0.63 0.83 0.76 |
| GEIM800111 | 27.8 94.7 60.1 60.6 15.5 68.7 68.2 24.5 50.7 22.8  27.6 103.0 33.5 25.5 51.5 42.0 45.0 34.7 55.2 23.7 |
| JANJ780101 | 51.0 5.0 22.0 19.0 74.0 16.0 16.0 52.0 34.0 66.0  60.0 3.0 52.0 58.0 25.0 35.0 30.0 49.0 24.0 64.0 |
| JANJ780102 | 15.0 67.0 49.0 50.0 5.0 56.0 55.0 10.0 34.0 13.0  16.0 85.0 20.0 10.0 45.0 32.0 32.0 17.0 41.0 14.0 |
| JANJ780103 | 1.7 0.1 0.4 0.4 4.6 0.3 0.3 1.8 0.8 3.1  2.4 0.05 1.9 2.2 0.6 0.8 0.7 1.6 0.5 2.9 |
| JANJ790101 | 0.3 -1.4 -0.5 -0.6 0.9 -0.7 -0.7 0.3 -0.1 0.7  0.5 -1.8 0.4 0.5 -0.3 -0.1 -0.2 0.3 -0.4 0.6 |
| JANJ790102 | 0.87 0.85 0.09 0.66 1.52 0.0 0.67 0.1 0.87 3.15  2.17 1.64 1.67 2.87 2.77 0.07 0.07 3.77 2.67 1.87 |
| JOND750101 | 2.34 1.18 2.02 2.01 1.65 2.17 2.19 2.34 1.82 2.36  2.36 2.18 2.28 1.83 1.99 2.21 2.1 2.38 2.2 2.32 |
| JOND750102 | 0.077 0.051 0.043 0.052 0.02 0.041 0.062 0.074 0.023 0.053  0.091 0.059 0.024 0.04 0.051 0.069 0.059 0.014 0.032 0.066 |
| JOND920101 | 100.0 83.0 104.0 86.0 44.0 84.0 77.0 50.0 91.0 103.0  54.0 72.0 93.0 51.0 58.0 117.0 107.0 25.0 50.0 98.0 |
| JOND920102 | 5.3 2.6 3.0 3.6 1.3 2.4 3.3 4.8 1.4 3.1  4.7 4.1 1.1 2.3 2.5 4.5 3.7 0.8 2.3 4.2 |
| JUKT750101 | 685.0 382.0 397.0 400.0 241.0 313.0 427.0 707.0 155.0 394.0  581.0 575.0 132.0 303.0 366.0 593.0 490.0 99.0 292.0 553.0 |
| JUNJ780101 | 1.36 1.0 0.89 1.04 0.82 1.14 1.48 0.63 1.11 1.08  1.21 1.22 1.45 1.05 0.52 0.74 0.81 0.97 0.79 0.94 |
| KANM800101 | 0.81 0.85 0.62 0.71 1.17 0.98 0.53 0.88 0.92 1.48  1.24 0.77 1.05 1.2 0.61 0.92 1.18 1.18 1.23 1.66 |
| KANM800102 | 1.45 1.15 0.64 0.91 0.7 1.14 1.29 0.53 1.13 1.23  1.56 1.27 1.83 1.2 0.21 0.48 0.77 1.17 0.74 1.1 |
| KANM800103 | 0.75 0.79 0.33 0.31 1.46 0.75 0.46 0.83 0.83 1.87  1.56 0.66 0.86 1.37 0.52 0.82 1.36 0.79 1.08 2.0 |
| KANM800104 | 1.041 1.038 1.117 1.033 0.96 1.165 1.094 1.142 0.982 1.002  0.967 1.093 0.947 0.93 1.055 1.169 1.073 0.925 0.961 0.982 |
| KARP850101 | 0.946 1.028 1.006 1.089 0.878 1.025 1.036 1.042 0.952 0.892  0.961 1.082 0.862 0.912 1.085 1.048 1.051 0.917 0.93 0.927 |
| KARP850102 | 0.892 0.901 0.93 0.932 0.925 0.885 0.933 0.923 0.894 0.872  0.921 1.057 0.804 0.914 0.932 0.923 0.934 0.803 0.837 0.913 |
| KARP850103 | 49.1 133.0 -3.6 0.0 0.0 20.0 0.0 64.6 75.7 18.9  15.6 0.0 6.8 54.7 43.8 44.4 31.0 70.5 0.0 29.5 |
| KHAG800101 | 0.0 1.0 0.0 -1.0 0.0 0.0 -1.0 0.0 0.0 0.0  0.0 1.0 0.0 0.0 0.0 0.0 0.0 0.0 0.0 0.0 |
| KRIW710101 | 4.6 6.5 5.9 5.7 -1.0 6.1 5.6 7.6 4.5 2.6  3.25 7.9 1.4 3.2 7.0 5.25 4.8 4.0 4.35 3.4 |
| KRIW790101 | 4.32 6.55 6.24 6.04 1.73 6.13 6.17 6.09 5.66 2.31  3.93 7.92 2.44 2.59 7.19 5.37 5.16 2.78 3.58 3.31 |
| KRIW790102 | 0.28 0.34 0.31 0.33 0.11 0.39 0.37 0.28 0.23 0.12  0.16 0.59 0.08 0.1 0.46 0.27 0.26 0.15 0.25 0.22 |
| KRIW790103 | 27.5 105.0 58.7 40.0 44.6 80.7 62.0 0.0 79.0 93.5  93.5 100.0 94.1 115.5 41.9 29.3 51.3 145.5 117.3 71.5 |
| KYTJ820101 | 1.8 -4.5 -3.5 -3.5 2.5 -3.5 -3.5 -0.4 -3.2 4.5  3.8 -3.9 1.9 2.8 -1.6 -0.8 -0.7 -0.9 -1.3 4.2 |
| LAWE840101 | -0.48 -0.06 -0.87 -0.75 -0.32 -0.32 -0.71 0.0 -0.51 0.81  1.02 -0.09 0.81 1.03 2.03 0.05 -0.35 0.66 1.24 0.56 |
| LEVM760101 | -0.5 3.0 0.2 2.5 -1.0 0.2 2.5 0.0 -0.5 -1.8  -1.8 3.0 -1.3 -2.5 -1.4 0.3 -0.4 -3.4 -2.3 -1.5 |
| LEVM760102 | 0.77 3.72 1.98 1.99 1.38 2.58 2.63 0.0 2.76 1.83  2.08 2.94 2.34 2.97 1.42 1.28 1.43 3.58 3.36 1.49 |
| LEVM760103 | 121.9 121.4 117.5 121.2 113.7 118.0 118.2 0.0 118.2 118.9  118.1 122.0 113.1 118.2 81.9 117.9 117.1 118.4 110.0 121.7 |
| LEVM760104 | 243.2 206.6 207.1 215.0 209.4 205.4 213.6 300.0 219.9 217.9  205.6 210.9 204.0 203.7 237.4 232.0 226.7 203.7 195.6 220.3 |
| LEVM760105 | 0.77 2.38 1.45 1.43 1.22 1.75 1.77 0.58 1.78 1.56  1.54 2.08 1.8 1.9 1.25 1.08 1.24 2.21 2.13 1.29 |
| LEVM760106 | 5.2 6.0 5.0 5.0 6.1 6.0 6.0 4.2 6.0 7.0  7.0 6.0 6.8 7.1 6.2 4.9 5.0 7.6 7.1 6.4 |
| LEVM760107 | 0.025 0.2 0.1 0.1 0.1 0.1 0.1 0.025 0.1 0.19  0.19 0.2 0.19 0.39 0.17 0.025 0.1 0.56 0.39 0.15 |
| LEVM780101 | 1.29 0.96 0.9 1.04 1.11 1.27 1.44 0.56 1.22 0.97  1.3 1.23 1.47 1.07 0.52 0.82 0.82 0.99 0.72 0.91 |
| LEVM780102 | 0.9 0.99 0.76 0.72 0.74 0.8 0.75 0.92 1.08 1.45  1.02 0.77 0.97 1.32 0.64 0.95 1.21 1.14 1.25 1.49 |
| LEVM780103 | 0.77 0.88 1.28 1.41 0.81 0.98 0.99 1.64 0.68 0.51  0.58 0.96 0.41 0.59 1.91 1.32 1.04 0.76 1.05 0.47 |
| LEVM780104 | 1.32 0.98 0.95 1.03 0.92 1.1 1.44 0.61 1.31 0.93  1.31 1.25 1.39 1.02 0.58 0.76 0.79 0.97 0.73 0.93 |
| LEVM780105 | 0.86 0.97 0.73 0.69 1.04 1.0 0.66 0.89 0.85 1.47  1.04 0.77 0.93 1.21 0.68 1.02 1.27 1.26 1.31 1.43 |
| LEVM780106 | 0.79 0.9 1.25 1.47 0.79 0.92 1.02 1.67 0.81 0.5  0.57 0.99 0.51 0.77 1.78 1.3 0.97 0.79 0.93 0.46 |
| LEWP710101 | 0.22 0.28 0.42 0.73 0.2 0.26 0.08 0.58 0.14 0.22  0.19 0.27 0.38 0.08 0.46 0.55 0.49 0.43 0.46 0.08 |
| LIFS790101 | 0.92 0.93 0.6 0.48 1.16 0.95 0.61 0.61 0.93 1.81  1.3 0.7 1.19 1.25 0.4 0.82 1.12 1.54 1.53 1.81 |
| LIFS790102 | 1.0 0.68 0.54 0.5 0.91 0.28 0.59 0.79 0.38 2.6  1.42 0.59 1.49 1.3 0.35 0.7 0.59 0.89 1.08 2.63 |
| LIFS790103 | 0.9 1.02 0.62 0.47 1.24 1.18 0.62 0.56 1.12 1.54  1.26 0.74 1.09 1.23 0.42 0.87 1.3 1.75 1.68 1.53 |
| MANP780101 | 12.97 11.72 11.42 10.85 14.63 11.76 11.89 12.43 12.16 15.67  14.9 11.36 14.39 14.0 11.37 11.23 11.69 13.93 13.42 15.71 |
| MAXF760101 | 1.43 1.18 0.64 0.92 0.94 1.22 1.67 0.46 0.98 1.04  1.36 1.27 1.53 1.19 0.49 0.7 0.78 1.01 0.69 0.98 |
| MAXF760102 | 0.86 0.94 0.74 0.72 1.17 0.89 0.62 0.97 1.06 1.24  0.98 0.79 1.08 1.16 1.22 1.04 1.18 1.07 1.25 1.33 |
| MAXF760103 | 0.64 0.62 3.14 1.92 0.32 0.8 1.01 0.63 2.05 0.92  0.37 0.89 1.07 0.86 0.5 1.01 0.92 1.0 1.31 0.87 |
| MAXF760104 | 0.17 0.76 2.62 1.08 0.95 0.91 0.28 5.02 0.57 0.26  0.21 1.17 0.0 0.28 0.12 0.57 0.23 0.0 0.97 0.24 |
| MAXF760105 | 1.13 0.48 1.11 1.18 0.38 0.41 1.02 3.84 0.3 0.4  0.65 1.13 0.0 0.45 0.0 0.81 0.71 0.93 0.38 0.48 |
| MAXF760106 | 1.0 1.18 0.87 1.39 1.09 1.13 1.04 0.46 0.71 0.68  1.01 1.05 0.36 0.65 1.95 1.56 1.23 1.1 0.87 0.58 |
| MCMT640101 | 4.34 26.66 13.28 12.0 35.77 17.56 17.26 0.0 21.81 19.06 18.78 21.29 21.64 29.4 10.93 6.35 11.01 42.53 31.53 13.92 |
| MEEJ800101 | 0.5 0.8 0.8 -8.2 -6.8 -4.8 -16.9 0.0 -3.5 13.9  8.8 0.1 4.8 13.2 6.1 1.2 2.7 14.9 6.1 2.7 |
| MEEJ800102 | -0.1 -4.5 -1.6 -2.8 -2.2 -2.5 -7.5 -0.5 0.8 11.8  10.0 -3.2 7.1 13.9 8.0 -3.7 1.5 18.1 8.2 3.3 |
| MEEJ810101 | 1.1 -0.4 -4.2 -1.6 7.1 -2.9 0.7 -0.2 -0.7 8.5  11.0 -1.9 5.4 13.4 4.4 -3.2 -1.7 17.1 7.4 5.9 |
| MEEJ810102 | 1.0 -2.0 -3.0 -0.5 4.6 -2.0 1.1 0.2 -2.2 7.0  9.6 -3.0 4.0 12.6 3.1 -2.9 -0.6 15.1 6.7 4.6 |
| MEIH800101 | 0.93 0.98 0.98 1.01 0.88 1.02 1.02 1.01 0.89 0.79  0.85 1.05 0.84 0.78 1.0 1.02 0.99 0.83 0.93 0.81 |
| MEIH800102 | 0.94 1.09 1.04 1.08 0.84 1.11 1.12 1.01 0.92 0.76  0.82 1.23 0.83 0.73 1.04 1.04 1.02 0.87 1.03 0.81 |
| MEIH800103 | 87.0 81.0 70.0 71.0 104.0 66.0 72.0 90.0 90.0 105.0  104.0 65.0 100.0 108.0 78.0 83.0 83.0 94.0 83.0 94.0 |
| MIYS850101 | 2.36 1.92 1.7 1.67 3.36 1.75 1.74 2.06 2.41 4.17 3.93 1.23 4.22 4.37 1.89 1.81 2.04 3.82 2.91 3.49 |
| NAGK730101 | 1.29 0.83 0.77 1.0 0.94 1.1 1.54 0.72 1.29 0.94 1.23 1.23 1.23 1.23 0.7 0.78 0.87 1.06 0.63 0.97 |
| NAGK730102 | 0.96 0.67 0.72 0.9 1.13 1.18 0.33 0.9 0.87 1.54 1.26 0.81 1.29 1.37 0.75 0.77 1.23 1.13 1.07 1.41 |
| NAGK730103 | 0.72 1.33 1.38 1.04 1.01 0.81 0.75 1.35 0.76 0.8 0.63 0.84 0.62 0.58 1.43 1.34 1.03 0.87 1.35 0.83 |
| NAKH900101 | 7.99 5.86 4.33 5.14 1.81 3.98 6.1 6.91 2.17 5.48 9.16 6.01 2.5 3.83 4.95 6.84 5.77 1.34 3.15 6.65 |
| NAKH900102 | 3.73 3.34 2.33 2.23 2.3 2.36 3.0 3.36 1.55 2.52 3.4 3.36 1.37 1.94 3.18 2.83 2.63 1.15 1.76 2.53 |
| NAKH900103 | 5.74 1.92 5.25 2.11 1.03 2.3 2.63 5.66 2.3 9.12  15.36 3.2 5.3 6.51 4.79 7.55 7.51 2.51 4.08 5.12 |
| NAKH900104 | -0.6 -1.18 0.39 -1.36 -0.34 -0.71 -1.16 -0.37 0.08 1.44 1.82 -0.84 2.04 1.38 -0.05 0.25 0.66 1.02 0.53 -0.6 |
| NAKH900105 | 5.88 1.54 4.38 1.7 1.11 2.3 2.6 5.29 2.33 8.78 16.52 2.58 6.0 6.58 5.29 7.68 8.38 2.89 3.51 4.66 |
| NAKH900106 | -0.57 -1.29 0.02 -1.54 -0.3 -0.71 -1.17 -0.48 0.1 1.31 2.16 -1.02 2.55 1.42 0.11 0.3 0.99 1.35 0.2 -0.79 |
| NAKH900107 | 5.39 2.81 7.31 3.07 0.86 2.31 2.7 6.52 2.23 9.94 12.64 4.67 3.68 6.34 3.62 7.24 5.44 1.64 5.42 6.18 |
| NAKH900108 | -0.7 -0.91 1.28 -0.93 -0.41 -0.71 -1.13 -0.12 0.04 1.77 1.02 -0.4 0.86 1.29 -0.42 0.14 -0.13 0.26 1.29 -0.19 |
| NAKH900109 | 9.25 3.96 3.71 3.89 1.07 3.17 4.8 8.51 1.88 6.47 10.94 3.5 3.14 6.36 4.36 6.26 5.66 2.22 3.28 7.55 |
| NAKH900110 | 0.34 -0.57 -0.27 -0.56 -0.32 -0.34 -0.43 0.48 -0.19 0.39 0.52 -0.75 0.47 1.3 -0.19 -0.2 -0.04 0.77 0.07 0.36 |
| NAKH900111 | 10.17 1.21 1.36 1.18 1.48 1.57 1.15 8.87 1.07 10.91 16.22 1.04 4.12 9.6 2.24 5.38 5.61 2.67 2.68 11.44 |
| NAKH900112 | 6.61 0.41 1.84 0.59 0.83 1.2 1.63 4.88 1.14 12.91 21.66 1.15 7.17 7.76 3.51 6.84 8.89 2.11 2.57 6.3 |
| NAKH900113 | 1.61 0.4 0.73 0.75 0.37 0.61 1.5 3.12 0.46 1.61 1.37 0.62 1.59 1.24 0.67 0.68 0.92 1.63 0.67 1.3 |
| NAKH920101 | 8.63 6.75 4.18 6.24 1.03 4.76 7.82 6.8 2.7 3.48 8.44 6.25 2.14 2.73 6.28 8.53 4.43 0.8 2.54 5.44 |
| NAKH920102 | 10.88 6.01 5.75 6.13 0.69 4.68 9.34 7.72 2.15 1.8 8.03 6.11 3.79 2.93 7.21 7.25 3.51 0.47 1.01 4.57 |
| NAKH920103 | 5.15 4.38 4.81 5.75 3.24 4.45 7.05 6.38 2.69 4.4 8.11 5.25 1.6 3.52 5.65 8.04 7.41 1.68 3.42 7.0 |
| NAKH920104 | 5.04 3.73 5.94 5.26 2.2 4.5 6.07 7.09 2.99 4.32 9.88 6.31 1.85 3.72 6.22 8.05 5.2 2.1 3.32 6.19 |
| NAKH920105 | 9.9 0.09 0.94 0.35 2.55 0.87 0.08 8.14 0.2 15.25 22.28 0.16 1.85 6.47 2.38 4.17 4.33 2.21 3.42 14.34 |
| NAKH920106 | 6.69 6.65 4.49 4.97 1.7 5.39 7.76 6.32 2.11 4.51 8.23 8.36 2.46 3.59 5.2 7.4 5.18 1.06 2.75 5.27 |
| NAKH920107 | 5.08 4.75 5.75 5.96 2.95 4.24 6.04 8.2 2.1 4.95 8.03 4.93 2.61 4.36 4.84 6.41 5.87 2.31 4.55 6.07 |
| NAKH920108 | 0.23 -0.26 -0.94 -1.13 1.78 -0.57 -0.75 -0.07 0.11 1.19 1.03 -1.05 0.66 0.48 -0.76 -0.67 -0.36 0.9 0.59 1.24 |
| NISK800101 | -0.22 -0.93 -2.65 -4.12 4.66 -2.76 -3.64 -1.62 1.28 5.58 5.01 -4.18 3.51 5.27 -3.03 -2.84 -1.2 5.2 2.15 4.45 |
| NISK860101 | 0.5 0.0 0.0 0.0 0.0 0.0 0.0 0.0 0.5 1.8 1.8 0.0 1.3 2.5 0.0 0.0 0.4 3.4 2.3 1.5 |
| NOZY710101 | -1.895 -1.475 -1.56 -1.518 -2.035 -1.521 -1.535 -1.898 -1.755 -1.951 -1.966 -1.374 -1.963 -1.864 -1.699 -1.753 -1.767 -1.869 -1.686 -1.981 |
| OOBM770101 | -1.404 -0.921 -1.178 -1.162 -1.365 -1.116 -1.163 -1.364 -1.215 -1.189 -1.315 -1.074 -1.303 -1.135 -1.236 -1.297 -1.252 -1.03 -1.03 -1.254 |
| OOBM770102 | -0.491 -0.554 -0.382 -0.356 -0.67 -0.405 -0.371 -0.534 -0.54 -0.762 -0.65 -0.3 -0.659 -0.729 -0.463 -0.455 -0.515 -0.839 -0.656 -0.728 |
| OOBM770103 | -9.475 -16.225 -12.48 -12.144 -12.21 -13.689 -13.815 -7.592 -17.55 -15.608 -15.728 -12.366 -15.704 -20.504 -11.893 -10.518 -12.369 -26.166 -20.232 -13.867 |
| OOBM770104 | -7.02 -10.131 -9.424 -9.296 -8.19 -10.044 -10.467 -5.456 -12.15 -9.512 -10.52 -9.666 -10.424 -12.485 -8.652 -7.782 -8.764 -14.42 -12.36 -8.778 |
| OOBM770105 | 2.01 0.84 0.03 -2.05 1.98 1.02 0.93 0.12 -0.14 3.7 2.73 2.55 1.75 2.68 0.41 1.47 2.39 2.49 2.23 3.5 |
| OOBM850101 | 1.34 0.95 2.49 3.32 1.07 1.49 2.2 2.07 1.27 0.66 0.54 0.61 0.7 0.8 2.12 0.94 1.09 -4.65 -0.17 1.32 |
| OOBM850102 | 0.46 -1.54 1.31 -0.33 0.2 -1.12 0.48 0.64 -1.31 3.28 0.43 -1.71 0.15 0.52 -0.58 -0.83 -1.52 1.25 -2.21 0.54 |
| OOBM850103 | -2.49 2.55 2.27 8.86 -3.13 1.79 4.04 -0.56 4.22 -10.87 -7.16 -9.97 -4.96 -6.64 5.19 -1.6 -4.75 -17.84 9.25 -3.97 |
| OOBM850104 | 4.55 5.97 5.56 2.85 -0.78 4.15 5.16 9.14 4.48 2.1 3.24 10.68 2.18 4.37 5.14 6.78 8.6 1.97 2.4 3.81 |
| OOBM850105 | 1.3 0.93 0.9 1.02 0.92 1.04 1.43 0.63 1.33 0.87 1.3 1.23 1.32 1.09 0.63 0.78 0.8 1.03 0.71 0.95 |
| PALJ810101 | 1.32 1.04 0.74 0.97 0.7 1.25 1.48 0.59 1.06 1.01 1.22 1.13 1.47 1.1 0.57 0.77 0.86 1.02 0.72 1.05 |
| PALJ810102 | 0.81 1.03 0.81 0.71 1.12 1.03 0.59 0.94 0.85 1.47 1.03 0.77 0.96 1.13 0.75 1.02 1.19 1.24 1.35 1.44 |
| PALJ810103 | 0.9 0.75 0.82 0.75 1.12 0.95 0.44 0.83 0.86 1.59 1.24 0.75 0.94 1.41 0.46 0.7 1.2 1.28 1.45 1.73 |
| PALJ810104 | 0.84 0.91 1.48 1.28 0.69 1.0 0.78 1.76 0.53 0.55 0.49 0.95 0.52 0.88 1.47 1.29 1.05 0.88 1.28 0.51 |
| PALJ810105 | 0.65 0.93 1.45 1.47 1.43 0.94 0.75 1.53 0.96 0.57 0.56 0.95 0.71 0.72 1.51 1.46 0.96 0.9 1.12 0.55 |
| PALJ810106 | 1.08 0.93 1.05 0.86 1.22 0.95 1.09 0.85 1.02 0.98 1.04 1.01 1.11 0.96 0.91 0.95 1.15 1.17 0.8 1.03 |
| PALJ810107 | 1.34 0.91 0.83 1.06 1.27 1.13 1.69 0.47 1.11 0.84 1.39 1.08 0.9 1.02 0.48 1.05 0.74 0.64 0.73 1.18 |
| PALJ810108 | 1.15 1.06 0.87 1.0 1.03 1.43 1.37 0.64 0.95 0.99 1.22 1.2 1.45 0.92 0.72 0.84 0.97 1.11 0.72 0.82 |
| PALJ810109 | 0.89 1.06 0.67 0.71 1.04 1.06 0.72 0.87 1.04 1.14 1.02 1.0 1.41 1.32 0.69 0.86 1.15 1.06 1.35 1.66 |
| PALJ810110 | 0.82 0.99 1.27 0.98 0.71 1.01 0.54 0.94 1.26 1.67 0.94 0.73 1.3 1.56 0.69 0.65 0.98 1.25 1.26 1.22 |
| PALJ810111 | 0.98 1.03 0.66 0.74 1.01 0.63 0.59 0.9 1.17 1.38 1.05 0.83 0.82 1.23 0.73 0.98 1.2 1.26 1.23 1.62 |
| PALJ810112 | 0.69 0.0 1.52 2.42 0.0 1.44 0.63 2.64 0.22 0.43 0.0 1.18 0.88 2.2 1.34 1.43 0.28 0.0 1.53 0.14 |
| PALJ810113 | 0.87 1.3 1.36 1.24 0.83 1.06 0.91 1.69 0.91 0.27 0.67 0.66 0.0 0.47 1.54 1.08 1.12 1.24 0.54 0.69 |
| PALJ810114 | 0.91 0.77 1.32 0.9 0.5 1.06 0.53 1.61 1.08 0.36 0.77 1.27 0.76 0.37 1.62 1.34 0.87 1.1 1.24 0.52 |
| PALJ810115 | 0.92 0.9 1.57 1.22 0.62 0.66 0.92 1.61 0.39 0.79 0.5 0.86 0.5 0.96 1.3 1.4 1.11 0.57 1.78 0.5 |
| PALJ810116 | 2.1 4.2 7.0 10.0 1.4 6.0 7.8 5.7 2.1 -8.0 -9.2 5.7 -4.2 -9.2 2.1 6.5 5.2 -10.0 -1.9 -3.7 |
| PARJ860101 | -2.89 -3.3 -3.41 -3.38 -2.49 -3.15 -2.94 -3.25 -2.84 -1.72 -1.61 -3.31 -1.84 -1.63 -2.5 -3.3 -2.91 -1.75 -2.42 -2.08 |
| PLIV810101 | 12.28 11.49 11.0 10.97 14.93 11.28 11.19 12.01 12.84 14.77 14.1 10.8 14.33 13.43 11.19 11.26 11.65 12.95 13.29 15.07 |
| PONP800101 | 7.62 6.81 6.17 6.18 10.93 6.67 6.38 7.31 7.85 9.99 9.37 5.72 9.83 8.99 6.64 6.93 7.08 8.41 8.53 10.38 |
| PONP800102 | 2.63 2.45 2.27 2.29 3.36 2.45 2.31 2.55 2.57 3.08 2.98 2.12 3.18 3.02 2.46 2.6 2.55 2.85 2.79 3.21 |
| PONP800103 | 13.65 11.28 12.24 10.98 14.49 11.3 12.55 15.36 11.59 14.63 14.01 11.96 13.4 14.08 11.51 11.26 13.0 12.06 12.64 12.88 |
| PONP800104 | 14.6 13.24 11.79 13.78 15.9 12.02 13.59 14.18 15.35 14.1 16.49 13.28 16.23 14.18 14.1 13.36 14.5 13.9 14.76 16.3 |
| PONP800105 | 10.67 11.05 10.85 10.21 14.15 11.71 11.71 10.95 12.07 12.95 13.07 9.93 15.0 13.27 10.62 11.18 10.53 11.41 11.52 13.86 |
| PONP800106 | 3.7 2.53 2.12 2.6 3.03 2.7 3.3 3.13 3.57 7.69 5.88 1.79 5.21 6.6 2.12 2.43 2.6 6.25 3.03 7.14 |
| PONP800107 | 6.05 5.7 5.04 4.95 7.86 5.45 5.1 6.16 5.8 7.51 7.37 4.88 6.39 6.62 5.65 5.53 5.81 6.98 6.73 7.62 |
| PONP800108 | 0.305 0.227 0.322 0.335 0.339 0.306 0.282 0.352 0.215 0.278 0.262 0.391 0.28 0.195 0.346 0.326 0.251 0.291 0.293 0.291 |
| PRAM820101 | 0.175 0.083 0.09 0.14 0.074 0.093 0.135 0.201 0.125 0.1 0.104 0.058 0.054 0.104 0.136 0.155 0.152 0.092 0.081 0.096 |
| PRAM820102 | 0.687 0.59 0.489 0.632 0.263 0.527 0.669 0.67 0.594 0.564 0.541 0.407 0.328 0.577 0.6 0.692 0.713 0.632 0.495 0.529 |
| PRAM820103 | -6.7 51.5 20.1 38.5 -8.4 17.2 34.3 -4.2 12.6 -13.0 -11.7 36.8 -14.2 -15.5 0.8 -2.5 -5.0 -7.9 2.9 -10.9 |
| PRAM900101 | 1.29 0.96 0.9 1.04 1.11 1.27 1.44 0.56 1.22 0.97 1.3 1.23 1.47 1.07 0.52 0.82 0.82 0.99 0.72 0.91 |
| PRAM900102 | 0.9 0.99 0.76 0.72 0.74 0.8 0.75 0.92 1.08 1.45 1.02 0.77 0.97 1.32 0.64 0.95 1.21 1.14 1.25 1.49 |
| PRAM900103 | 0.78 0.88 1.28 1.41 0.8 0.97 1.0 1.64 0.69 0.51 0.59 0.96 0.39 0.58 1.91 1.33 1.03 0.75 1.05 0.47 |
| PRAM900104 | 1.0 0.7 0.6 0.5 1.9 1.0 0.7 0.3 0.8 4.0 2.0 0.7 1.9 3.1 0.2 0.9 1.7 2.2 2.8 4.0 |
| PTIO830101 | 0.12 0.04 -0.1 0.01 -0.25 -0.03 -0.02 -0.02 -0.06 -0.07 0.05 0.26 0.0 0.05 -0.19 -0.19 -0.04 -0.06 -0.14 -0.03 |
| PTIO830102 | 0.26 -0.14 -0.03 0.15 -0.15 -0.13 0.21 -0.37 0.1 -0.03 -0.02 0.12 0.0 0.12 -0.08 0.01 -0.34 -0.01 -0.29 0.02 |
| QIAN880101 | 0.64 -0.1 0.09 0.33 0.03 -0.23 0.51 -0.09 -0.23 -0.22 0.41 -0.17 0.13 -0.03 -0.43 -0.1 -0.07 -0.02 -0.38 -0.01 |
| QIAN880102 | 0.29 -0.03 -0.04 0.11 -0.05 0.26 0.28 -0.67 -0.26 0.0 0.47 -0.19 0.27 0.24 -0.34 -0.17 -0.2 0.25 -0.3 -0.01 |
| QIAN880103 | 0.68 -0.22 -0.09 -0.02 -0.15 -0.15 0.44 -0.73 -0.14 -0.08 0.61 0.03 0.39 0.06 -0.76 -0.26 -0.1 0.2 -0.04 0.12 |
| QIAN880104 | 0.34 0.22 -0.33 0.06 -0.18 0.01 0.2 -0.88 -0.09 -0.03 0.2 -0.11 0.43 0.15 -0.81 -0.35 -0.37 0.07 -0.31 0.13 |
| QIAN880105 | 0.57 0.23 -0.36 -0.46 -0.15 0.15 0.26 -0.71 -0.05 0.0 0.48 0.16 0.41 0.03 -1.12 -0.47 -0.54 -0.1 -0.35 0.31 |
| QIAN880106 | 0.33 0.1 -0.19 -0.44 -0.03 0.19 0.21 -0.46 0.27 -0.33 0.57 0.23 0.79 0.48 -1.86 -0.23 -0.33 0.15 -0.19 0.24 |
| QIAN880107 | 0.13 0.08 -0.07 -0.71 -0.09 0.12 0.13 -0.39 0.32 0.0 0.5 0.37 0.63 0.15 -1.4 -0.28 -0.21 0.02 -0.1 0.17 |
| QIAN880108 | 0.31 0.18 -0.1 -0.81 -0.26 0.41 -0.06 -0.42 0.51 -0.15 0.56 0.47 0.58 0.1 -1.33 -0.49 -0.44 0.14 -0.08 -0.01 |
| QIAN880109 | 0.21 0.07 -0.04 -0.58 -0.12 0.13 -0.23 -0.15 0.37 0.31 0.7 0.28 0.61 -0.06 -1.03 -0.28 -0.25 0.21 0.16 0.0 |
| QIAN880110 | 0.18 0.21 -0.03 -0.32 -0.29 -0.27 -0.25 -0.4 0.28 -0.03 0.62 0.41 0.21 0.05 -0.84 -0.05 -0.16 0.32 0.11 0.06 |
| QIAN880111 | -0.08 0.05 -0.08 -0.24 -0.25 -0.28 -0.19 -0.1 0.29 -0.01 0.28 0.45 0.11 0.0 -0.42 0.07 -0.33 0.36 0.0 -0.13 |
| QIAN880112 | -0.18 -0.13 0.28 0.05 -0.26 0.21 -0.06 0.23 0.24 -0.42 -0.23 0.03 -0.42 -0.18 -0.13 0.41 0.33 -0.1 -0.1 -0.07 |
| QIAN880113 | -0.01 0.02 0.41 -0.09 -0.27 0.01 0.09 0.13 0.22 -0.27 -0.25 0.08 -0.57 -0.12 0.26 0.44 0.35 -0.15 0.15 -0.09 |
| QIAN880114 | -0.19 0.03 0.02 -0.06 -0.29 0.02 -0.1 0.19 -0.16 -0.08 -0.42 -0.09 -0.38 -0.32 0.05 0.25 0.22 -0.19 0.05 -0.15 |
| QIAN880115 | -0.14 0.14 -0.27 -0.1 -0.64 -0.11 -0.39 0.46 -0.04 0.16 -0.57 0.04 0.24 0.08 0.02 -0.12 0.0 -0.1 0.18 0.29 |
| QIAN880116 | -0.31 0.25 -0.53 -0.54 -0.06 0.07 -0.52 0.37 -0.32 0.57 0.09 -0.29 0.29 0.24 -0.31 0.11 0.03 0.15 0.29 0.48 |
| QIAN880117 | -0.1 0.19 -0.89 -0.89 0.13 -0.04 -0.34 -0.45 -0.34 0.95 0.32 -0.46 0.43 0.36 -0.91 -0.12 0.49 0.34 0.42 0.76 |
| QIAN880118 | -0.25 -0.02 -0.77 -1.01 0.13 -0.12 -0.62 -0.72 -0.16 1.1 0.23 -0.59 0.32 0.48 -1.24 -0.31 0.17 0.45 0.77 0.69 |
| QIAN880119 | -0.26 -0.09 -0.34 -0.55 0.47 -0.33 -0.75 -0.56 -0.04 0.94 0.25 -0.55 -0.05 0.2 -1.28 -0.28 0.08 0.22 0.53 0.67 |
| QIAN880120 | 0.05 -0.11 -0.4 -0.11 0.36 -0.67 -0.35 0.14 0.02 0.47 0.32 -0.51 -0.1 0.2 -0.79 0.03 -0.15 0.09 0.34 0.58 |
| QIAN880121 | -0.44 -0.13 0.05 -0.2 0.13 -0.58 -0.28 0.08 0.09 -0.04 -0.12 -0.33 -0.21 -0.13 -0.48 0.27 0.47 -0.22 -0.11 0.06 |
| QIAN880122 | -0.31 -0.1 0.06 0.13 -0.11 -0.47 -0.05 0.45 -0.06 -0.25 -0.44 -0.44 -0.28 -0.04 -0.29 0.34 0.27 -0.08 0.06 0.11 |
| QIAN880123 | -0.02 0.04 0.03 0.11 -0.02 -0.17 0.1 0.38 -0.09 -0.48 -0.26 -0.39 -0.14 -0.03 -0.04 0.41 0.36 -0.01 -0.08 -0.18 |
| QIAN880124 | -0.06 0.02 0.1 0.24 -0.19 -0.04 -0.04 0.17 0.19 -0.2 -0.46 -0.43 -0.52 -0.33 0.37 0.43 0.5 -0.32 0.35 0.0 |
| QIAN880125 | -0.05 0.06 0.0 0.15 0.3 -0.08 -0.02 -0.14 -0.07 0.26 0.04 -0.42 0.25 0.09 0.31 -0.11 -0.06 0.19 0.33 0.04 |
| QIAN880126 | -0.19 0.17 -0.38 0.09 0.41 0.04 -0.2 0.28 -0.19 -0.06 0.34 -0.2 0.45 0.07 0.04 -0.23 -0.02 0.16 0.22 0.05 |
| QIAN880127 | -0.43 0.06 0.0 -0.31 0.19 0.14 -0.41 -0.21 0.21 0.29 -0.1 0.33 -0.01 0.25 0.28 -0.23 -0.26 0.15 0.09 -0.1 |
| QIAN880128 | -0.19 -0.07 0.17 -0.27 0.42 -0.29 -0.22 0.17 0.17 -0.34 -0.22 0.0 -0.53 -0.31 0.14 0.22 0.1 -0.15 -0.02 -0.33 |
| QIAN880129 | -0.25 0.12 0.61 0.6 0.18 0.09 -0.12 0.09 0.42 -0.54 -0.55 0.14 -0.47 -0.29 0.89 0.24 0.16 -0.44 -0.19 -0.45 |
| QIAN880130 | -0.27 -0.4 0.71 0.54 0.0 -0.08 -0.12 1.14 0.18 -0.74 -0.54 0.45 -0.76 -0.47 1.4 0.4 -0.1 -0.46 -0.05 -0.86 |
| QIAN880131 | -0.42 -0.23 0.81 0.95 -0.18 -0.01 -0.09 1.24 0.05 -1.17 -0.69 0.09 -0.86 -0.39 1.77 0.63 0.29 -0.37 -0.41 -1.32 |
| QIAN880132 | -0.24 -0.04 0.45 0.65 -0.38 0.01 0.07 0.85 -0.21 -0.65 -0.8 0.17 -0.71 -0.61 2.27 0.33 0.13 -0.44 -0.49 -0.99 |
| QIAN880133 | -0.14 0.21 0.35 0.66 -0.09 0.11 0.06 0.36 -0.31 -0.51 -0.8 -0.14 -0.56 -0.25 1.59 0.32 0.21 -0.17 -0.35 -0.7 |
| QIAN880134 | 0.01 -0.13 -0.11 0.78 -0.31 -0.13 0.09 0.14 -0.56 -0.09 -0.81 -0.43 -0.49 -0.2 1.14 0.13 -0.02 -0.2 0.1 -0.11 |
| QIAN880135 | -0.3 -0.09 -0.12 0.44 0.03 0.24 0.18 -0.12 -0.2 -0.07 -0.18 0.06 -0.44 0.11 0.77 -0.09 -0.27 -0.09 -0.25 -0.06 |
| QIAN880136 | -0.23 -0.2 0.06 0.34 0.19 0.47 0.28 0.14 -0.22 0.42 -0.36 -0.15 -0.19 -0.02 0.78 -0.29 -0.3 -0.18 0.07 0.29 |
| QIAN880137 | 0.08 -0.01 -0.06 0.04 0.37 0.48 0.36 -0.02 -0.45 0.09 0.24 -0.27 0.16 0.34 0.16 -0.35 -0.04 -0.06 -0.2 0.18 |
| QIAN880138 | 0.934 0.962 0.986 0.994 0.9 1.047 0.986 1.015 0.882 0.766 0.825 1.04 0.804 0.773 1.047 1.056 1.008 0.848 0.931 0.825 |
| QIAN880139 | 0.941 1.112 1.038 1.071 0.866 1.15 1.1 1.055 0.911 0.742 0.798 1.232 0.781 0.723 1.093 1.082 1.043 0.867 1.05 0.817 |
| RACS770101 | 1.16 1.72 1.97 2.66 0.5 3.87 2.4 1.63 0.86 0.57 0.51 3.9 0.4 0.43 2.04 1.61 1.48 0.75 1.72 0.59 |
| RACS770102 | 0.85 2.02 0.88 1.5 0.9 1.71 1.79 1.54 1.59 0.67 1.03 0.88 1.17 0.85 1.47 1.5 1.96 0.83 1.34 0.89 |
| RACS770103 | 1.58 1.14 0.77 0.98 1.04 1.24 1.49 0.66 0.99 1.09 1.21 1.27 1.41 1.0 1.46 1.05 0.87 1.23 0.68 0.88 |
| RACS820101 | 0.82 2.6 2.07 2.64 0.0 0.0 2.62 1.63 0.0 2.32 0.0 2.86 0.0 0.0 0.0 1.23 2.48 0.0 1.9 1.62 |
| RACS820102 | 0.78 1.75 1.32 1.25 3.14 0.93 0.94 1.13 1.03 1.26 0.91 0.85 0.41 1.07 1.73 1.31 1.57 0.98 1.31 1.11 |
| RACS820103 | 0.88 0.99 1.02 1.16 1.14 0.93 1.01 0.7 1.87 1.61 1.09 0.83 1.71 1.52 0.87 1.14 0.96 1.96 1.68 1.56 |
| RACS820104 | 0.3 0.9 2.73 1.26 0.72 0.97 1.33 3.09 1.33 0.45 0.96 0.71 1.89 1.2 0.83 1.16 0.97 1.58 0.86 0.64 |
| RACS820105 | 0.4 1.2 1.24 1.59 2.98 0.5 1.26 1.89 2.71 1.31 0.57 0.87 0.0 1.27 0.38 0.92 1.38 1.53 1.79 0.95 |
| RACS820106 | 1.48 1.02 0.99 1.19 0.86 1.42 1.43 0.46 1.27 1.12 1.33 1.36 1.41 1.3 0.25 0.89 0.81 1.27 0.91 0.93 |
| RACS820107 | 0.0 0.0 4.14 2.15 0.0 0.0 0.0 6.49 0.0 0.0 0.0 0.0 0.0 2.11 1.99 0.0 1.24 0.0 1.9 0.0 |
| RACS820108 | 1.02 1.0 1.31 1.76 1.05 1.05 0.83 2.39 0.4 0.83 1.06 0.94 1.33 0.41 2.73 1.18 0.77 1.22 1.09 0.88 |
| RACS820109 | 0.93 1.52 0.92 0.6 1.08 0.94 0.73 0.78 1.08 1.74 1.03 1.0 1.31 1.51 1.37 0.97 1.38 1.12 1.65 1.7 |
| RACS820110 | 0.99 1.19 1.15 1.18 2.32 1.52 1.36 1.4 1.06 0.81 1.26 0.91 1.0 1.25 0.0 1.5 1.18 1.33 1.09 1.01 |
| RACS820111 | 17.05 21.25 34.81 19.27 28.84 15.42 20.12 38.14 23.07 16.66 10.89 16.46 20.61 16.26 23.94 19.95 18.92 23.36 26.49 17.06 |
| RACS820112 | 14.53 17.82 13.59 19.78 30.57 22.18 18.19 37.16 22.63 20.28 14.3 14.07 20.61 19.61 52.63 18.56 21.09 19.78 26.36 21.87 |
| RACS820113 | 1.81 -14.92 -6.64 -8.72 1.28 -5.54 -6.81 0.94 -4.66 4.92 4.92 -5.55 2.35 2.98 0.0 -3.4 -2.57 2.33 -0.14 4.04 |
| RACS820114 | 0.52 -1.32 -0.01 0.0 0.0 -0.07 -0.79 0.0 0.95 2.04 1.76 0.08 1.32 2.09 0.0 0.04 0.27 2.51 1.63 1.18 |
| RADA880101 | 0.13 -5.0 -3.04 -2.23 -2.52 -3.84 -3.43 1.45 -5.61 -2.77 -2.64 -3.97 -3.83 -3.74 0.0 -1.66 -2.31 -8.21 -5.97 -2.05 |
| RADA880102 | 1.29 -13.6 -6.63 0.0 0.0 -5.47 -6.02 0.94 -5.61 2.88 3.16 -5.63 1.03 0.89 0.0 -3.44 -2.84 -0.18 -1.77 2.86 |
| RADA880103 | 1.42 -18.6 -9.67 0.0 0.0 -9.31 -9.45 2.39 -11.22 0.11 0.52 -9.6 -2.8 -2.85 0.0 -5.1 -5.15 -8.39 -7.74 0.81 |
| RADA880104 | 93.7 250.4 146.3 142.6 135.2 177.7 182.9 52.6 188.1 182.2 173.7 215.2 197.6 228.6 0.0 109.5 142.1 271.6 239.9 157.2 |
| RADA880105 | -0.29 -2.71 -1.18 -1.02 0.0 -1.53 -0.9 -0.34 -0.94 0.24 -0.12 -2.05 -0.24 0.0 0.0 -0.75 -0.71 -0.59 -1.02 0.09 |
| RADA880106 | -0.06 -0.84 -0.48 -0.8 1.36 -0.73 -0.77 -0.41 0.49 1.31 1.21 -1.18 1.27 1.27 0.0 -0.5 -0.27 0.88 0.33 1.09 |
| RADA880107 | 0.7 0.4 1.2 1.4 0.6 1.0 1.0 1.6 1.2 0.9 0.9 1.0 0.3 1.2 0.7 1.6 0.3 1.1 1.9 0.7 |
| RADA880108 | 0.7 0.4 1.2 1.4 0.6 1.0 1.0 1.6 1.2 0.9 0.9 1.0 0.3 1.2 0.7 1.6 0.3 1.1 1.9 0.7 |
| RICJ880101 | 0.5 0.4 3.5 2.1 0.6 0.4 0.4 1.8 1.1 0.2 0.2 0.7 0.8 0.2 0.8 2.3 1.6 0.3 0.8 0.1 |
| RICJ880102 | 1.2 0.7 0.7 0.8 0.8 0.7 2.2 0.3 0.7 0.9 0.9 0.6 0.3 0.5 2.6 0.7 0.8 2.1 1.8 1.1 |
| RICJ880103 | 1.6 0.9 0.7 2.6 1.2 0.8 2.0 0.9 0.7 0.7 0.3 1.0 1.0 0.9 0.5 0.8 0.7 1.7 0.4 0.6 |
| RICJ880104 | 1.0 0.4 0.7 2.2 0.6 1.5 3.3 0.6 0.7 0.4 0.6 0.8 1.0 0.6 0.4 0.4 1.0 1.4 1.2 1.1 |
| RICJ880105 | 1.1 1.5 0.0 0.3 1.1 1.3 0.5 0.4 1.5 1.1 2.6 0.8 1.7 1.9 0.1 0.4 0.5 3.1 0.6 1.5 |
| RICJ880106 | 1.4 1.2 1.2 0.6 1.6 1.4 0.9 0.6 0.9 0.9 1.1 1.9 1.7 1.0 0.3 1.1 0.6 1.4 0.2 0.8 |
| RICJ880107 | 1.8 1.3 0.9 1.0 0.7 1.3 0.8 0.5 1.0 1.2 1.2 1.1 1.5 1.3 0.3 0.6 1.0 1.5 0.8 1.2 |
| RICJ880108 | 1.8 1.0 0.6 0.7 0.0 1.0 1.1 0.5 2.4 1.3 1.2 1.4 2.7 1.9 0.3 0.5 0.5 1.1 1.3 0.4 |
| RICJ880109 | 1.3 0.8 0.6 0.5 0.7 0.2 0.7 0.5 1.9 1.6 1.4 1.0 2.8 2.9 0.0 0.5 0.6 2.1 0.8 1.4 |
| RICJ880110 | 0.7 0.8 0.8 0.6 0.2 1.3 1.6 0.1 1.1 1.4 1.9 2.2 1.0 1.8 0.0 0.6 0.7 0.4 1.1 1.3 |
| RICJ880111 | 1.4 2.1 0.9 0.7 1.2 1.6 1.7 0.2 1.8 0.4 0.8 1.9 1.3 0.3 0.2 1.6 0.9 0.4 0.3 0.7 |
| RICJ880112 | 1.1 1.0 1.2 0.4 1.6 2.1 0.8 0.2 3.4 0.7 0.7 2.0 1.0 0.7 0.0 1.7 1.0 0.0 1.2 0.7 |
| RICJ880113 | 0.8 0.9 1.6 0.7 0.4 0.9 0.3 3.9 1.3 0.7 0.7 1.3 0.8 0.5 0.7 0.8 0.3 0.0 0.8 0.2 |
| RICJ880114 | 1.0 1.4 0.9 1.4 0.8 1.4 0.8 1.2 1.2 1.1 0.9 1.2 0.8 0.1 1.9 0.7 0.8 0.4 0.9 0.6 |
| RICJ880115 | 0.7 1.1 1.5 1.4 0.4 1.1 0.7 0.6 1.0 0.7 0.5 1.3 0.0 1.2 1.5 0.9 2.1 2.7 0.5 1.0 |
| RICJ880116 | 6.5 -0.9 -5.1 0.5 -1.3 1.0 7.8 -8.6 1.2 0.6 3.2 2.3 5.3 1.6 -7.7 -3.9 -2.6 1.2 -4.5 1.4 |
| RICJ880117 | 2.3 -5.2 0.3 7.4 0.8 -0.7 10.3 -5.2 -2.8 -4.0 -2.1 -4.1 -3.5 -1.1 8.1 -3.5 2.3 -0.9 -3.7 -4.4 |
| ROBB760101 | 6.7 0.3 -6.1 -3.1 -4.9 0.6 2.2 -6.8 -1.0 3.2 5.5 0.5 7.2 2.8 -22.8 -3.0 -4.0 4.0 -4.6 2.5 |
| ROBB760102 | 2.3 1.4 -3.3 -4.4 6.1 2.7 2.5 -8.3 5.9 -0.5 0.1 7.3 3.5 1.6 -24.4 -1.9 -3.7 -0.9 -0.6 2.3 |
| ROBB760103 | -2.3 0.4 -4.1 -4.4 4.4 1.2 -5.0 -4.2 -2.5 6.7 2.3 -3.3 2.3 2.6 -1.8 -1.7 1.3 -1.0 4.0 6.8 |
| ROBB760104 | -2.7 0.4 -4.2 -4.4 3.7 0.8 -8.1 -3.9 -3.0 7.7 3.7 -2.9 3.7 3.0 -6.6 -2.4 1.7 0.3 3.3 7.1 |
| ROBB760105 | 0.0 1.1 -2.0 -2.6 5.4 2.4 3.1 -3.4 0.8 -0.1 3.7 -3.1 -2.1 0.7 7.4 1.3 0.0 -3.4 4.8 2.7 |
| ROBB760106 | -5.0 2.1 4.2 3.1 4.4 0.4 -4.7 5.7 -0.3 -4.6 -5.6 1.0 -4.8 -1.8 2.6 2.6 0.3 3.4 2.9 -6.0 |
| ROBB760107 | -3.3 0.0 5.4 3.9 -0.3 -0.4 -1.8 -1.2 3.0 -0.5 -2.3 -1.2 -4.3 0.8 6.5 1.8 -0.7 -0.8 3.1 -3.5 |
| ROBB760108 | -4.7 2.0 3.9 1.9 6.2 -2.0 -4.2 5.7 -2.6 -7.0 -6.2 2.8 -4.8 -3.7 3.6 2.1 0.6 3.3 3.8 -6.2 |
| ROBB760109 | -3.7 1.0 -0.6 -0.6 4.0 3.4 -4.3 5.9 -0.8 -0.5 -2.8 1.3 -1.6 1.6 -6.0 1.5 1.2 6.5 1.3 -4.6 |
| ROBB760110 | -2.5 -1.2 4.6 0.0 -4.7 -0.5 -4.4 4.9 1.6 -3.3 -2.0 -0.8 -4.1 -4.1 5.8 2.5 1.7 1.2 -0.6 -3.5 |
| ROBB760111 | -5.1 2.6 4.7 3.1 3.8 0.2 -5.2 5.6 -0.9 -4.5 -5.4 1.0 -5.3 -2.4 3.5 3.2 0.0 2.9 3.2 -6.3 |
| ROBB760112 | -1.0 0.3 -0.7 -1.2 2.1 -0.1 -0.7 0.3 1.1 4.0 2.0 -0.9 1.8 2.8 0.4 -1.2 -0.5 3.0 2.1 1.4 |
| ROBB760113 | 86.6 162.2 103.3 97.8 132.3 119.2 113.9 62.9 155.8 158.0 164.1 115.5 172.9 194.1 92.9 85.6 106.5 224.6 177.7 141.0 |
| ROBB790101 | 0.74 0.64 0.63 0.62 0.91 0.62 0.62 0.72 0.78 0.88 0.85 0.52 0.85 0.88 0.64 0.66 0.7 0.85 0.76 0.86 |
| ROSG850101 | -0.67 12.1 7.23 8.72 -0.34 6.39 7.35 0.0 3.82 -3.02 -3.02 6.13 -1.3 -3.24 -1.75 4.35 3.86 -2.86 0.98 -2.18 |
| ROSG850102 | -0.67 3.89 2.27 1.57 -2.0 2.12 1.78 0.0 1.09 -3.02 -3.02 2.46 -1.67 -3.24 -1.75 0.1 -0.42 -2.86 0.98 -2.18 |
| ROSM880101 | 0.4 0.3 0.9 0.8 0.5 0.7 1.3 0.0 1.0 0.4 0.6 0.4 0.3 0.7 0.9 0.4 0.4 0.6 1.2 0.4 |
| ROSM880102 | 0.73 0.73 -0.01 0.54 0.7 -0.1 0.55 0.0 1.1 2.97 2.49 1.5 1.3 2.65 2.6 0.04 0.44 3.0 2.97 1.69 |
| ROSM880103 | 0.239 0.211 0.249 0.171 0.22 0.26 0.187 0.16 0.205 0.273 0.281 0.228 0.253 0.234 0.165 0.236 0.213 0.183 0.193 0.255 |
| SIMZ760101 | 0.33 -0.176 -0.233 -0.371 0.074 -0.254 -0.409 0.37 -0.078 0.149 0.129 -0.075 -0.092 -0.011 0.37 0.022 0.136 -0.011 -0.138 0.245 |
| SNEP660101 | -0.11 0.079 -0.136 -0.285 -0.184 -0.067 -0.246 -0.073 0.32 0.001 -0.008 0.049 -0.041 0.438 -0.016 -0.153 -0.208 0.493 0.381 -0.155 |
| SNEP660102 | -0.062 -0.167 0.166 -0.079 0.38 -0.025 -0.184 -0.017 0.056 -0.309 -0.264 -0.371 0.077 0.074 -0.036 0.47 0.348 0.05 0.22 -0.212 |
| SNEP660103 | 1.071 1.033 0.784 0.68 0.922 0.977 0.97 0.591 0.85 1.14 1.14 0.939 1.2 1.086 0.659 0.76 0.817 1.107 1.02 0.95 |
| SNEP660104 | 8.0 0.1 0.1 70.0 26.0 33.0 6.0 0.1 0.1 55.0 33.0 1.0 54.0 18.0 42.0 0.1 0.1 77.0 66.0 0.1 |
| SUEM840101 | -0.4 -0.59 -0.92 -1.31 0.17 -0.91 -1.22 -0.67 -0.64 1.25 1.22 -0.67 1.02 1.92 -0.49 -0.55 -0.28 0.5 1.67 0.91 |
| SUEM840102 | 1.42 1.06 0.71 1.01 0.73 1.02 1.63 0.5 1.2 1.12 1.29 1.24 1.21 1.16 0.65 0.71 0.78 1.05 0.67 0.99 |
| SWER830101 | 0.946 1.128 0.432 1.311 0.481 1.615 0.698 0.36 2.168 1.283 1.192 1.203 0.0 0.963 2.093 0.523 1.961 1.925 0.802 0.409 |
| TANS770101 | 0.79 1.087 0.832 0.53 1.268 1.038 0.643 0.725 0.864 1.361 1.111 0.735 1.092 1.052 1.249 1.093 1.214 1.114 1.34 1.428 |
| TANS770102 | 1.194 0.795 0.659 1.056 0.678 1.29 0.928 1.015 0.611 0.603 0.595 1.06 0.831 0.377 3.159 1.444 1.172 0.452 0.816 0.64 |
| TANS770103 | 0.497 0.677 2.072 1.498 1.348 0.711 0.651 1.848 1.474 0.471 0.656 0.932 0.425 1.348 0.179 1.151 0.749 1.283 1.283 0.654 |
| TANS770104 | 0.937 1.725 1.08 1.64 1.004 1.078 0.679 0.901 1.085 0.178 0.808 1.254 0.886 0.803 0.748 1.145 1.487 0.803 1.227 0.625 |
| TANS770105 | 0.289 1.38 3.169 0.917 1.767 2.372 0.285 4.259 1.061 0.262 0.0 1.288 0.0 0.393 0.0 0.16 0.218 0.0 0.654 0.167 |
| TANS770106 | 0.328 2.088 1.498 3.379 0.0 0.0 0.0 0.5 1.204 2.078 0.414 0.835 0.982 1.336 0.415 1.089 1.732 1.781 0.0 0.946 |
| TANS770107 | 0.945 0.364 1.202 1.315 0.932 0.704 1.014 2.355 0.525 0.673 0.758 0.947 1.028 0.622 0.579 1.14 0.863 0.777 0.907 0.561 |
| TANS770108 | 0.842 0.936 1.352 1.366 1.032 0.998 0.758 1.349 1.079 0.459 0.665 1.045 0.668 0.881 1.385 1.257 1.055 0.881 1.101 0.643 |
| TANS770109 | 0.135 0.296 0.196 0.289 0.159 0.236 0.184 0.051 0.223 0.173 0.215 0.17 0.239 0.087 0.151 0.01 0.1 0.166 0.066 0.285 |
| TANS770110 | 0.507 0.459 0.287 0.223 0.592 0.383 0.445 0.39 0.31 0.111 0.619 0.559 0.431 0.077 0.739 0.689 0.785 0.16 0.06 0.356 |
| VASM830101 | 0.159 0.194 0.385 0.283 0.187 0.236 0.206 0.049 0.233 0.581 0.083 0.159 0.198 0.682 0.366 0.15 0.074 0.463 0.737 0.301 |
| VASM830102 | 0.03731 0.09593 0.00359 0.1263 0.08292 0.07606 0.0058 0.00499 0.02415 0.0 0.0 0.0371 0.08226 0.0946 0.01979 0.08292 0.09408 0.05481 0.05159 0.00569 |
| VASM830103 | 0.0 0.0 0.0 0.0 0.0 0.0 0.0 0.0 0.0 1.0 1.0 0.0 0.0 1.0 0.0 0.0 0.0 1.0 1.0 1.0 |
| VELV850101 | -12.04 39.23 4.25 23.22 3.95 2.16 16.81 -7.85 6.28 -18.32 -17.79 9.71 -8.86 -21.98 5.82 -1.54 -4.15 -16.19 -1.51 -16.22 |
| VENT840101 | 10.04 6.18 5.63 5.76 8.89 5.41 5.37 7.99 7.49 8.72 8.79 4.4 9.15 7.98 7.79 7.08 7.0 8.07 6.9 8.88 |
| VHEG790101 | 0.89 0.88 0.89 0.87 0.85 0.82 0.84 0.92 0.83 0.76 0.73 0.97 0.74 0.52 0.82 0.96 0.92 0.2 0.49 0.85 |
| WARP780101 | 0.52 0.49 0.42 0.37 0.83 0.35 0.38 0.41 0.7 0.79 0.77 0.31 0.76 0.87 0.35 0.49 0.38 0.86 0.64 0.72 |
| WEBA780101 | 0.16 -0.2 1.03 -0.24 -0.12 -0.55 -0.45 -0.16 -0.18 -0.19 -0.44 -0.12 -0.79 -0.25 -0.59 -0.01 0.05 -0.33 -0.42 -0.46 |
| WERD780101 | 0.15 -0.37 0.69 -0.22 -0.19 -0.06 0.14 0.36 -0.25 0.02 0.06 -0.16 0.11 1.18 0.11 0.13 0.28 -0.12 0.19 -0.08 |
| WERD780102 | -0.07 -0.4 -0.57 -0.8 0.17 -0.26 -0.63 0.27 -0.49 0.06 -0.17 -0.45 0.03 0.4 -0.47 -0.11 0.09 -0.61 -0.61 -0.11 |
| WERD780103 | 7.0 9.1 10.0 13.0 5.5 8.6 12.5 7.9 8.4 4.9 4.9 10.1 5.3 5.0 6.6 7.5 6.6 5.3 5.7 5.6 |
| WERD780104 | 1.94 -19.92 -9.68 -10.95 -1.24 -9.38 -10.2 2.39 -10.27 2.15 2.28 -9.52 -1.48 -0.76 -3.68 -5.06 -4.88 -5.88 -6.11 1.99 |
| WOEC730101 | 0.07 2.88 3.22 3.64 0.71 2.18 3.08 2.23 2.41 -4.44 -4.19 2.84 -2.49 -4.92 -1.22 1.96 0.92 -4.75 -1.39 -2.69 |
| WOLR810101 | -1.73 2.52 1.45 1.13 -0.97 0.53 0.39 -5.36 1.74 -1.68 -1.03 1.41 -0.27 1.3 0.88 -1.63 -2.09 3.65 2.32 -2.53 |
| WOLS870101 | 0.09 -3.44 0.84 2.36 4.13 -1.14 -0.07 0.3 1.11 -1.03 -0.98 -3.14 -0.41 0.45 2.23 0.57 -1.4 0.85 0.01 -1.29 |
| WOLS870102 | 8.5 0.0 8.2 8.5 11.0 6.3 8.8 7.1 10.1 16.8 15.0 7.9 13.3 11.2 8.2 7.4 8.8 9.9 8.8 12.0 |
| WOLS870103 | 6.8 0.0 6.2 7.0 8.3 8.5 4.9 6.4 9.2 10.0 12.2 7.5 8.4 8.3 6.9 8.0 7.0 5.7 6.8 9.4 |
| YUTK870101 | 18.08 0.0 17.47 17.36 18.17 17.93 18.16 18.24 18.49 18.62 18.6 17.96 18.11 17.3 18.16 17.57 17.54 17.19 17.99 18.3 |
| YUTK870102 | 18.56 0.0 18.24 17.94 17.84 18.51 17.97 18.57 18.64 19.21 19.01 18.36 18.49 17.95 18.77 18.06 17.71 16.87 18.23 18.98 |
| YUTK870103 | -0.152 -0.089 -0.203 -0.355 0.0 -0.181 -0.411 -0.19 0.0 -0.086 -0.102 -0.062 -0.107 0.001 -0.181 -0.203 -0.17 0.275 0.0 -0.125 |
| YUTK870104 | 0.83 0.83 0.09 0.64 1.48 0.0 0.65 0.1 1.1 3.07 2.52 1.6 1.4 2.75 2.7 0.14 0.54 0.31 2.97 1.79 |
| ZASB820101 | 11.5 14.28 12.82 11.68 13.46 14.45 13.57 3.4 13.69 21.4 21.4 15.71 16.25 19.8 17.43 9.47 15.77 21.67 18.03 21.57 |
| ZIMJ680101 | 0.0 52.0 3.38 49.7 1.48 3.53 49.9 0.0 51.6 0.13 0.13 49.5 1.43 0.35 1.58 1.67 1.66 2.1 1.61 0.13 |
| ZIMJ680102 | 6.0 10.76 5.41 2.77 5.05 5.65 3.22 5.97 7.59 6.02 5.98 9.74 5.74 5.48 6.3 5.68 5.66 5.89 5.66 5.96 |
| ZIMJ680103 | 9.9 4.6 5.4 2.8 2.8 9.0 3.2 5.6 8.2 17.1 17.6 3.5 14.9 18.8 14.8 6.9 9.5 17.1 15.0 14.3 |
| ZIMJ680104 | 0.94 1.15 0.79 1.19 0.6 0.94 1.41 1.18 1.15 1.07 0.95 1.03 0.88 1.06 1.18 0.69 0.87 0.91 1.04 0.9 |
| ZIMJ680105 | 0.98 1.14 1.05 1.05 0.41 0.9 1.04 1.25 1.01 0.88 0.8 1.06 1.12 1.12 1.31 1.02 0.8 0.9 1.12 0.87 |
| AURR980101 | 1.05 0.81 0.91 1.39 0.6 0.87 1.11 1.26 1.43 0.95 0.96 0.97 0.99 0.95 1.05 0.96 1.03 1.06 0.94 0.62 |
| AURR980102 | 0.75 0.9 1.24 1.72 0.66 1.08 1.1 1.14 0.96 0.8 1.01 0.66 1.02 0.88 1.33 1.2 1.13 0.68 0.8 0.58 |
| AURR980103 | 0.67 0.76 1.28 1.58 0.37 1.05 0.94 0.98 0.83 0.78 0.79 0.84 0.98 0.96 1.12 1.25 1.41 0.94 0.82 0.67 |
| AURR980104 | 1.1 1.05 0.72 1.14 0.26 1.31 2.3 0.55 0.83 1.06 0.84 1.08 0.9 0.9 1.67 0.81 0.77 1.26 0.99 0.76 |
| AURR980105 | 1.39 0.95 0.67 1.64 0.52 1.6 2.07 0.65 1.36 0.64 0.91 0.8 1.1 1.0 0.94 0.69 0.92 1.1 0.73 0.7 |
| AURR980106 | 1.43 1.33 0.55 0.9 0.52 1.43 1.7 0.56 0.66 1.18 1.52 0.82 1.68 1.1 0.15 0.61 0.75 1.68 0.65 1.14 |
| AURR980107 | 1.55 1.39 0.6 0.61 0.59 1.43 1.34 0.37 0.89 1.47 1.36 1.27 2.13 1.39 0.03 0.44 0.65 1.1 0.93 1.18 |
| AURR980108 | 1.8 1.73 0.73 0.9 0.55 0.97 1.73 0.32 0.46 1.09 1.47 1.24 1.64 0.96 0.15 0.67 0.7 0.68 0.91 0.81 |
| AURR980109 | 1.52 1.49 0.58 1.04 0.26 1.41 1.76 0.3 0.83 1.25 1.26 1.1 1.14 1.14 0.44 0.66 0.73 0.68 1.04 1.03 |
| AURR980110 | 1.49 1.41 0.67 0.94 0.37 1.52 1.55 0.29 0.96 1.04 1.4 1.17 1.84 0.86 0.2 0.68 0.79 1.52 1.06 0.94 |
| AURR980111 | 1.73 1.24 0.7 0.68 0.63 0.88 1.16 0.32 0.76 1.15 1.8 1.22 2.21 1.35 0.07 0.65 0.46 1.57 1.1 0.94 |
| AURR980112 | 1.33 1.39 0.64 0.6 0.44 1.37 1.43 0.2 1.02 1.58 1.63 1.71 1.76 1.22 0.07 0.42 0.57 1.0 1.02 1.08 |
| AURR980113 | 1.87 1.66 0.7 0.91 0.33 1.24 1.88 0.33 0.89 0.9 1.65 1.63 1.35 0.67 0.03 0.71 0.5 1.0 0.73 0.51 |
| AURR980114 | 1.19 1.45 1.33 0.72 0.44 1.43 1.27 0.74 1.55 0.61 1.36 1.45 1.35 1.2 0.1 1.02 0.82 0.58 1.06 0.46 |
| AURR980115 | 0.77 1.11 1.39 0.79 0.44 0.95 0.92 2.74 1.65 0.64 0.66 1.19 0.74 1.04 0.66 0.64 0.82 0.58 0.93 0.53 |
| AURR980116 | 0.93 0.96 0.82 1.15 0.67 1.02 1.07 1.08 1.4 1.14 1.16 1.27 1.11 1.05 1.01 0.71 0.84 1.06 1.15 0.74 |
| AURR980117 | 1.09 1.29 1.03 1.17 0.26 1.08 1.31 0.97 0.88 0.97 0.87 1.13 0.96 0.84 2.01 0.76 0.79 0.91 0.64 0.77 |
| AURR980118 | 0.71 1.09 0.95 1.43 0.65 0.87 1.19 1.07 1.13 1.05 0.84 1.1 0.8 0.95 1.7 0.65 0.086 1.25 0.85 1.12 |
| AURR980119 | 13.4 13.3 12.0 11.7 11.6 12.8 12.2 11.3 11.6 12.0 13.0 13.0 12.8 12.1 6.5 12.2 11.7 12.4 12.1 11.9 |
| AURR980120 | -0.77 -0.68 -0.07 -0.15 -0.23 -0.33 -0.27 0.0 -0.06 -0.23 -0.62 -0.65 -0.5 -0.41 3.0 -0.35 -0.11 -0.45 -0.17 -0.14 |
| ONEK900101 | 0.984 1.008 1.048 1.068 0.906 1.037 1.094 1.031 0.95 0.927 0.935 1.102 0.952 0.915 1.049 1.046 0.997 0.904 0.929 0.931 |
| ONEK900102 | 1.315 1.31 1.38 1.372 1.196 1.342 1.376 1.382 1.279 1.241 1.234 1.367 1.269 1.247 1.342 1.381 1.324 1.186 1.199 1.235 |
| VINM940101 | 0.994 1.026 1.022 1.022 0.939 1.041 1.052 1.018 0.967 0.977 0.982 1.029 0.963 0.934 1.05 1.025 0.998 0.938 0.981 0.968 |
| VINM940102 | 0.783 0.807 0.799 0.822 0.785 0.817 0.826 0.784 0.777 0.776 0.783 0.834 0.806 0.774 0.809 0.811 0.795 0.796 0.788 0.781 |
| VINM940103 | 0.423 0.503 0.906 0.87 0.877 0.594 0.167 1.162 0.802 0.566 0.494 0.615 0.444 0.706 1.945 0.928 0.884 0.69 0.778 0.706 |
| VINM940104 | 0.619 0.753 1.089 0.932 1.107 0.77 0.675 1.361 1.034 0.876 0.74 0.784 0.736 0.968 1.78 0.969 1.053 0.91 1.009 0.939 |
| MUNV940101 | 1.08 0.976 1.197 1.266 0.733 1.05 1.085 1.104 0.906 0.583 0.789 1.026 0.812 0.685 1.412 0.987 0.784 0.755 0.665 0.546 |
| MUNV940102 | 0.978 0.784 0.915 1.038 0.573 0.863 0.962 1.405 0.724 0.502 0.766 0.841 0.729 0.585 2.613 0.784 0.569 0.671 0.56 0.444 |
| MUNV940103 | 1.4 1.23 1.61 1.89 1.14 1.33 1.42 2.06 1.25 1.02 1.33 1.34 1.12 1.07 3.9 1.2 0.99 1.1 0.98 0.87 |
| MUNV940104 | 4.08 3.91 3.83 3.02 4.49 3.67 2.23 4.24 4.08 4.52 4.81 3.77 4.48 5.38 3.8 4.12 4.11 6.1 5.19 4.18 |
| MUNV940105 | -0.35 -0.44 -0.38 -0.41 -0.47 -0.4 -0.41 0.0 -0.46 -0.56 -0.48 -0.41 -0.46 -0.55 -0.23 -0.39 -0.48 -0.48 -0.5 -0.53 |
| WIMW960101 | 0.5 1.7 1.7 1.6 0.6 1.6 1.6 1.3 1.6 0.6 0.4 1.6 0.5 0.4 1.7 0.7 0.4 0.7 0.6 0.5 |
| KIMC930101 | 0.96 0.77 0.39 0.42 0.42 0.8 0.53 0.0 0.57 0.84 0.92 0.73 0.86 0.59 -2.5 0.53 0.54 0.58 0.72 0.63 |
| MONM990101 | 0.343 0.353 0.409 0.429 0.319 0.395 0.405 0.389 0.307 0.296 0.287 0.429 0.293 0.292 0.432 0.416 0.362 0.268 0.22 0.307 |
| BLAM930101 | 0.32 0.327 0.384 0.424 0.198 0.436 0.514 0.374 0.299 0.306 0.34 0.446 0.313 0.314 0.354 0.376 0.339 0.291 0.287 0.294 |
| PARS000101 | 8.9 4.6 4.4 6.3 0.6 2.8 6.9 9.4 2.2 7.0 7.4 6.1 2.3 3.3 4.2 4.0 5.7 1.3 4.5 8.2 |
| PARS000102 | 9.2 3.6 5.1 6.0 1.0 2.9 6.0 9.4 2.1 6.0 7.7 6.5 2.4 3.4 4.2 5.5 5.7 1.2 3.7 8.2 |
| KUMS000101 | 14.1 5.5 3.2 5.7 0.1 3.7 8.8 4.1 2.0 7.1 9.1 7.7 3.3 5.0 0.7 3.9 4.4 1.2 4.5 5.9 |
| KUMS000102 | 13.4 3.9 3.7 4.6 0.8 4.8 7.8 4.6 3.3 6.5 10.6 7.5 3.0 4.5 1.3 3.8 4.6 1.0 3.3 7.1 |
| KUMS000103 | 9.8 7.3 3.6 4.9 3.0 2.4 4.4 0.0 11.9 17.2 17.0 10.5 11.9 23.0 15.0 2.6 6.9 24.2 17.2 15.3 |
| KUMS000104 | 0.7 0.95 1.47 0.87 1.17 0.73 0.96 0.64 1.39 1.29 1.44 0.91 0.91 1.34 0.12 0.84 0.74 1.8 1.68 1.2 |
| TAKK010101 | 58.0 -184.0 -93.0 -97.0 116.0 -139.0 -131.0 -11.0 -73.0 107.0 95.0 -24.0 78.0 92.0 -79.0 -34.0 -7.0 59.0 -11.0 100.0 |
| FODM020101 | 51.0 -144.0 -84.0 -78.0 137.0 -128.0 -115.0 -13.0 -55.0 106.0 103.0 -205.0 73.0 108.0 -79.0 -26.0 -3.0 69.0 11.0 108.0 |
| NADH010101 | 32.0 -95.0 -73.0 -29.0 182.0 -95.0 -74.0 -22.0 -25.0 106.0 104.0 -124.0 82.0 132.0 -82.0 -34.0 20.0 118.0 44.0 113.0 |
| NADH010102 | 24.0 -79.0 -76.0 0.0 194.0 -87.0 -57.0 -28.0 -31.0 102.0 103.0 -9.0 90.0 131.0 -85.0 -36.0 34.0 116.0 43.0 111.0 |
| NADH010103 | 5.0 -57.0 -77.0 45.0 224.0 -67.0 -8.0 -47.0 -50.0 83.0 82.0 -38.0 83.0 117.0 -103.0 -41.0 79.0 130.0 27.0 117.0 |
| NADH010104 | -2.0 -41.0 -97.0 248.0 329.0 -37.0 117.0 -66.0 -70.0 28.0 36.0 115.0 62.0 120.0 -132.0 -52.0 174.0 179.0 -7.0 114.0 |
| NADH010105 | 0.4 1.5 1.6 1.5 0.7 1.4 1.3 1.1 1.4 0.5 0.3 1.4 0.5 0.3 1.6 0.9 0.7 0.9 0.9 0.4 |
| NADH010106 | -0.04 -0.3 0.25 0.27 0.57 -0.02 -0.33 1.24 -0.11 -0.26 -0.38 -0.18 -0.09 -0.01 0.0 0.15 0.39 0.21 0.05 -0.06 |
| NADH010107 | -0.12 0.34 1.05 1.12 -0.63 1.67 0.91 0.76 1.34 -0.77 0.15 0.29 -0.71 -0.67 0.0 1.45 -0.7 -0.14 -0.49 -0.7 |
| MONM990201 | 8.6 4.2 4.6 4.9 2.9 4.0 5.1 7.8 2.1 4.6 8.8 6.3 2.5 3.7 4.9 7.3 6.0 1.4 3.6 6.7 |
| KOEP990101 | 7.6 5.0 4.4 5.2 2.2 4.1 6.2 6.9 2.1 5.1 9.4 5.8 2.1 4.0 5.4 7.2 6.1 1.4 3.2 6.7 |
| KOEP990102 | 8.1 4.6 3.7 3.8 2.0 3.1 4.6 7.0 2.0 6.7 11.0 4.4 2.8 5.6 4.7 7.3 5.6 1.8 3.3 7.7 |
| CEDJ970101 | 7.9 4.9 4.0 5.5 1.9 4.4 7.1 7.1 2.1 5.2 8.6 6.7 2.4 3.9 5.3 6.6 5.3 1.2 3.1 6.8 |
| CEDJ970102 | 8.3 8.7 3.7 4.7 1.6 4.7 6.5 6.3 2.1 3.7 7.4 7.9 2.3 2.7 6.9 8.8 5.1 0.7 2.4 5.3 |
| CEDJ970103 | 4.47 8.48 3.89 7.05 0.29 2.87 16.56 8.29 1.74 3.3 5.06 12.98 1.71 2.32 5.41 4.27 3.83 0.67 2.75 4.05 |
| CEDJ970104 | 6.77 6.87 5.5 8.57 0.31 5.24 12.93 7.95 2.8 2.72 4.43 10.2 1.87 1.92 4.79 5.41 5.36 0.54 2.26 3.57 |
| CEDJ970105 | 7.43 4.51 9.12 8.71 0.42 5.42 5.86 9.4 1.49 1.76 2.74 9.67 0.6 1.18 5.6 9.6 8.95 1.18 3.26 3.1 |
| FUKS010101 | 5.22 7.3 6.06 7.91 1.01 6.0 10.66 5.81 2.27 2.36 4.52 12.68 1.85 1.68 5.7 6.99 5.16 0.56 2.16 4.1 |
| FUKS010102 | 9.88 3.71 2.35 3.5 1.12 1.66 4.02 6.88 1.88 10.08 13.21 3.39 2.44 5.27 3.8 4.1 4.98 1.11 4.07 12.53 |
| FUKS010103 | 10.98 3.26 2.85 3.37 1.47 2.3 3.51 7.48 2.2 9.74 12.79 2.54 3.1 4.97 3.42 4.93 5.55 1.28 3.55 10.69 |
| FUKS010104 | 9.95 3.05 4.84 4.46 1.3 2.64 2.58 8.87 1.99 7.73 9.66 2.0 2.45 5.41 3.2 6.03 5.62 2.6 6.15 9.46 |
| FUKS010105 | 8.26 2.8 2.54 2.8 2.67 2.86 2.67 5.62 1.98 8.95 16.46 1.89 2.67 7.32 3.3 6.0 5.0 2.01 3.96 10.24 |
| FUKS010106 | 7.39 5.91 3.06 5.14 0.74 2.22 9.8 7.53 1.82 6.96 9.45 7.81 2.1 3.91 4.54 4.18 4.45 0.9 3.46 8.62 |
| FUKS010107 | 9.07 4.9 4.05 5.73 0.95 3.63 7.77 7.69 2.47 6.56 9.0 6.01 2.54 3.59 4.04 5.15 5.46 0.95 2.96 7.47 |
| FUKS010108 | 8.82 3.71 6.77 6.38 0.9 3.89 4.05 9.11 1.77 5.05 6.54 5.45 1.62 3.51 4.28 7.64 7.12 1.96 4.85 6.6 |
| FUKS010109 | 6.65 5.17 4.4 5.5 1.79 4.52 6.89 5.72 2.13 5.47 10.15 7.59 2.24 4.34 4.56 6.52 5.08 1.24 3.01 7.0 |
| FUKS010110 | 0.163 0.22 0.124 0.212 0.316 0.274 0.212 0.08 0.315 0.474 0.315 0.255 0.356 0.41 NaN 0.29 0.412 0.325 0.354 0.515 |
| FUKS010111 | 0.236 0.233 0.189 0.168 0.259 0.314 0.306 -0.17 0.256 0.391 0.293 0.231 0.367 0.328 NaN 0.202 0.308 0.197 0.223 0.436 |
| FUKS010112 | -0.49 -0.429 -0.387 -0.375 -0.352 -0.422 -0.382 -0.647 -0.357 -0.268 -0.45 -0.409 -0.375 -0.309 NaN -0.426 -0.24 -0.325 -0.288 -0.22 |
| AVBF000101 | -0.871 -0.727 -0.741 -0.737 -0.666 -0.728 -0.773 -0.822 -0.685 -0.617 -0.798 -0.715 -0.717 -0.649 NaN -0.679 -0.629 -0.669 -0.655 -0.599 |
| AVBF000102 | -0.393 -0.317 -0.268 -0.247 -0.222 -0.291 -0.26 -0.57 -0.244 -0.144 -0.281 -0.294 -0.274 -0.189 NaN -0.28 -0.152 -0.206 -0.155 -0.08 |
| AVBF000103 | -0.378 -0.369 -0.245 -0.113 -0.206 -0.29 -0.165 -0.56 -0.295 -0.134 -0.266 -0.335 -0.26 -0.187 NaN -0.251 -0.093 -0.188 -0.147 -0.084 |
| AVBF000104 | -0.729 -0.535 -0.597 -0.545 -0.408 -0.492 -0.532 -0.86 -0.519 -0.361 -0.462 -0.508 -0.518 -0.454 NaN -0.278 -0.367 -0.455 -0.439 -0.323 |
| AVBF000105 | -0.623 -0.567 -0.619 -0.626 -0.571 -0.559 -0.572 -0.679 -0.508 -0.199 -0.527 -0.581 -0.571 -0.461 NaN -0.458 -0.233 -0.327 -0.451 -0.263 |
| AVBF000106 | -0.376 -0.28 -0.403 -0.405 -0.441 -0.362 -0.362 -0.392 -0.345 -0.194 -0.317 -0.412 -0.312 -0.237 NaN -0.374 -0.243 -0.111 -0.171 -0.355 |
| AVBF000107 | NaN 0.62 0.76 0.66 0.83 0.59 0.73 NaN 0.92 0.88 0.89 0.77 0.77 0.92 0.94 0.58 0.73 0.86 0.93 0.88 |
| AVBF000108 | 0.0 2.45 0.0 0.0 0.0 1.25 1.27 0.0 1.45 0.0 0.0 3.67 0.0 0.0 0.0 0.0 0.0 6.93 5.06 0.0 |
| AVBF000109 | 89.3 190.3 122.4 114.4 102.5 146.9 138.8 63.8 157.5 163.0 163.1 165.1 165.8 190.8 121.6 94.2 119.6 226.4 194.6 138.2 |
| YANJ020101 | 90.0 194.0 124.7 117.3 103.3 149.4 142.2 64.9 160.0 163.9 164.0 167.3 167.0 191.9 122.9 95.4 121.5 228.2 197.0 139.0 |
| MITS020101 | 0.0373 0.0959 0.0036 0.1263 0.0829 0.0761 0.0058 0.005 0.0242 0.0 0.0 0.0371 0.0823 0.0946 0.0198 0.0829 0.0941 0.0548 0.0516 0.0057 |
| TSAJ990101 | 0.85 0.2 -0.48 -1.1 2.1 -0.42 -0.79 0.0 0.22 3.14 1.99 -1.19 1.42 1.69 -1.14 -0.52 -0.08 1.76 1.37 2.53 |
| TSAJ990102 | 0.06 -0.85 0.25 -0.2 0.49 0.31 -0.1 0.21 -2.24 3.48 3.5 -1.62 0.21 4.8 0.71 -0.62 0.65 2.29 1.89 1.59 |
| COSI940101 | 2.62 1.26 -1.27 -2.84 0.73 -1.69 -0.4 -1.15 -0.74 4.38 6.57 -2.78 -3.12 9.14 -0.12 -1.39 1.81 5.91 1.39 2.3 |
| PONP930101 | -1.64 -3.28 0.83 0.7 9.3 -0.04 1.18 -1.85 7.17 3.02 0.83 -2.36 4.26 -1.36 3.12 1.59 2.31 2.61 2.37 0.52 |
| WILM950101 | -2.34 1.6 2.81 -0.48 5.03 0.16 1.3 -1.06 -3.0 7.26 1.09 1.56 0.62 2.57 -0.15 1.93 0.19 3.59 -2.58 2.06 |
| WILM950102 | 0.78 1.58 1.2 1.35 0.55 1.19 1.45 0.68 0.99 0.47 0.56 1.1 0.66 0.47 0.69 1.0 1.05 0.7 1.0 0.51 |
| WILM950103 | 1.1 -5.1 -3.5 -3.6 2.5 -3.68 -3.2 -0.64 -3.2 4.5 3.8 -4.11 1.9 2.8 -1.9 -0.5 -0.7 -0.46 -1.3 4.2 |
| WILM950104 | 0.1366 0.0363 -0.0345 -0.1233 0.2745 0.0325 -0.0484 -0.0464 0.0549 0.4172 0.4251 -0.0101 0.1747 0.4076 0.0019 -0.0433 0.0589 0.2362 0.3167 0.4084 |
| KUHL950101 | 0.0728 0.0394 -0.039 -0.0552 0.3557 0.0126 -0.0295 -0.0589 0.0874 0.3805 0.3819 -0.0053 0.1613 0.4201 -0.0492 -0.0282 0.0239 0.4114 0.3113 0.2947 |
| GUOD860101 | 0.151 -0.0103 0.0381 0.0047 0.3222 0.0246 -0.0639 0.0248 0.1335 0.4238 0.3926 -0.0158 0.216 0.3455 0.0844 0.004 0.1462 0.2657 0.2998 0.3997 |
| JURD980101 | -0.058 0.0 0.027 0.016 0.447 -0.073 -0.128 0.331 0.195 0.06 0.138 -0.112 0.275 0.24 -0.478 -0.177 -0.163 0.564 0.322 -0.052 |
| BASU050101 | -0.17 0.37 0.18 0.37 -0.06 0.26 0.15 0.01 -0.02 -0.28 -0.28 0.32 -0.26 -0.41 0.13 0.05 0.02 -0.15 -0.09 -0.17 |
| BASU050102 | -0.15 0.32 0.22 0.41 -0.15 0.03 0.3 0.08 0.06 -0.29 -0.36 0.24 -0.19 -0.22 0.15 0.16 -0.08 -0.28 -0.03 -0.24 |
| BASU050103 | 0.964 1.143 0.944 0.916 0.778 1.047 1.051 0.835 1.014 0.922 1.085 0.944 1.032 1.119 1.299 0.947 1.017 0.895 1.0 0.955 |
| SUYM030101 | 0.974 1.129 0.988 0.892 0.972 1.092 1.054 0.845 0.949 0.928 1.11 0.946 0.923 1.122 1.362 0.932 1.023 0.879 0.902 0.923 |
| PUNT030101 | 0.938 1.137 0.902 0.857 0.6856 0.916 1.139 0.892 1.109 0.986 1.0 0.952 1.077 1.11 1.266 0.956 1.018 0.971 1.157 0.959 |
| PUNT030102 | 1.042 1.069 0.828 0.97 0.5 1.111 0.992 0.743 1.034 0.852 1.193 0.979 0.998 0.981 1.332 0.984 0.992 0.96 1.12 1.001 |
| GEOR030101 | 1.065 1.131 0.762 0.836 1.015 0.861 0.736 1.022 0.973 1.189 1.192 0.478 1.369 1.368 1.241 1.097 0.822 1.017 0.836 1.14 |
| GEOR030102 | 0.99 1.132 0.873 0.915 0.644 0.999 1.053 0.785 1.054 0.95 1.106 1.003 1.093 1.121 1.314 0.911 0.988 0.939 1.09 0.957 |
| GEOR030103 | 0.892 1.154 1.144 0.925 1.035 1.2 1.115 0.917 0.992 0.817 0.994 0.944 0.782 1.058 1.309 0.986 1.11 0.841 0.866 0.9 |
| GEOR030104 | 1.092 1.239 0.927 0.919 0.662 1.124 1.199 0.698 1.012 0.912 1.276 1.008 1.171 1.09 0.8 0.886 0.832 0.981 1.075 0.908 |
| GEOR030105 | 0.843 1.038 0.956 0.906 0.896 0.968 0.9 0.978 1.05 0.946 0.885 0.893 0.878 1.151 1.816 1.003 1.189 0.852 0.945 0.999 |
| GEOR030106 | 2.18 2.71 1.85 1.75 3.89 2.16 1.89 1.17 2.51 4.5 4.71 2.12 3.63 5.88 2.09 1.66 2.18 6.46 5.01 3.77 |
| GEOR030107 | 1.79 3.2 2.83 2.33 2.22 2.37 2.52 0.7 3.06 4.59 4.72 2.5 3.91 4.84 2.45 1.82 2.45 5.64 4.46 3.67 |
| GEOR030108 | 13.4 8.5 7.6 8.2 22.6 8.5 7.3 7.0 11.3 20.3 20.8 6.1 15.7 23.9 9.9 8.2 10.3 24.5 19.5 19.5 |
| GEOR030109 | 0.0166 -0.0762 -0.0786 -0.1278 0.5724 -0.1051 -0.1794 -0.0442 0.1643 0.2758 0.2523 -0.2134 0.0197 0.3561 -0.4188 -0.1629 -0.0701 0.3836 0.25 0.1782 |
| ZHOH040101 | 90.1 192.8 127.5 117.1 113.2 149.4 140.8 63.8 159.3 164.9 164.6 170.0 167.7 193.5 123.1 94.2 120.0 197.1 231.7 139.1 |
| ZHOH040102 | 91.5 196.1 138.3 135.2 114.4 156.4 154.6 67.5 163.2 162.6 163.4 162.5 165.9 198.8 123.4 102.0 126.0 209.8 237.2 138.4 |
| ZHOH040103 | 1.076 1.361 1.056 1.29 0.753 0.729 1.118 1.346 0.985 0.926 1.054 1.105 0.974 0.869 0.82 1.342 0.871 0.666 0.531 1.131 |
| BAEK050101 | 1.12 -2.55 -0.83 -0.83 0.59 -0.78 -0.92 1.2 -0.93 1.16 1.18 -0.8 0.55 0.67 0.54 -0.05 -0.02 -0.19 -0.23 1.13 |
| HARY940101 | 1.38 0.0 0.37 0.52 1.43 0.22 0.71 1.34 0.66 2.32 1.47 0.15 1.78 1.72 0.85 0.86 0.89 0.82 0.47 1.99 |
| PONJ960101 | -0.27 1.87 0.81 0.81 -1.05 1.1 1.17 -0.16 0.28 -0.77 -1.1 1.7 -0.73 -1.43 -0.75 0.42 0.63 -1.57 -0.56 -0.4 |
| DIGM050101 | 0.05 0.12 0.29 0.41 -0.84 0.46 0.38 0.31 -0.41 -0.69 -0.62 0.57 -0.38 -0.45 0.46 0.12 0.38 -0.98 -0.25 -0.46 |
| WOLR790101 | 0.54 -0.16 0.38 0.65 -1.13 0.05 0.38 NaN -0.59 -2.15 -1.08 0.48 -0.97 -1.51 -0.22 0.65 0.27 -1.61 -1.13 -0.75 |
| OLSK800101 | -0.31 1.3 0.49 0.58 -0.87 0.7 0.68 -0.33 0.13 -0.66 -0.53 1.79 -0.38 -0.45 0.34 0.1 0.21 -0.27 0.4 -0.62 |
| KIDA850101 | -0.27 2.0 0.61 0.5 -0.23 1.0 0.33 -0.22 0.37 -0.8 -0.44 1.17 -0.31 -0.55 0.36 0.17 0.18 0.05 0.48 -0.65 |
| GUYH850102 | 0.39 NaN -1.91 -0.71 0.25 -1.3 -0.18 0.0 -0.6 1.82 1.82 0.32 0.96 2.27 NaN -1.24 -1.0 2.13 1.47 1.3 |
| GUYH850103 | 0.39 -3.95 -1.91 -3.81 0.25 -1.3 -2.91 0.0 -0.64 1.82 1.82 -2.77 0.96 2.27 NaN -1.24 -1.0 2.13 1.47 1.3 |
| GUYH850104 | 0.18 -5.4 -1.3 -2.36 0.27 -1.22 -2.1 0.09 -1.48 0.37 0.41 -2.53 0.44 0.5 -0.2 -0.4 -0.34 -0.01 -0.08 0.32 |
| GUYH850105 | 0.42 -1.56 -1.03 -0.51 0.84 -0.96 -0.37 0.0 -2.28 1.81 1.8 -2.03 1.18 1.74 0.86 -0.64 -0.26 1.46 0.51 1.34 |
| ROSM880104 | 0.616 0.0 0.236 0.028 0.68 0.251 0.043 0.501 0.165 0.943 0.943 0.283 0.738 1.0 0.711 0.359 0.45 0.878 0.88 0.825 |
| ROSM880105 | 0.2 -0.7 -0.5 -1.4 1.9 -1.1 -1.3 -0.1 0.4 1.4 0.5 -1.6 0.5 1.0 -1.0 -0.7 -0.4 1.6 0.5 0.7 |
| JACR890101 | 50.76 48.66 45.8 43.17 58.74 46.09 43.48 50.27 49.33 57.3 53.89 42.92 52.75 53.45 45.39 47.24 49.26 53.59 51.79 56.12 |
| COWR900101 | -0.414 -0.584 -0.916 -1.31 0.162 -0.905 -1.218 -0.684 -0.63 1.237 1.215 -0.67 1.02 1.938 -0.503 -0.563 -0.289 0.514 1.699 0.899 |
| BLAS910101 | -0.96 0.75 -1.94 -5.68 4.54 -5.3 -3.86 -1.28 -0.62 5.54 6.81 -5.62 4.76 5.06 -4.47 -1.92 -3.99 0.21 3.34 5.39 |
| CASG920101 | -0.26 0.08 -0.46 -1.3 0.83 -0.83 -0.73 -0.4 -0.18 1.1 1.52 -1.01 1.09 1.09 -0.62 -0.55 -0.71 -0.13 0.69 1.15 |
| CORJ870101 | -0.73 -1.03 -5.29 -6.13 0.64 -0.96 -2.9 -2.67 3.03 5.04 4.91 -5.99 3.34 5.2 -4.32 -3.0 -1.91 0.51 2.87 3.98 |
| CORJ870102 | -1.35 -3.89 -10.96 -11.88 4.37 -1.34 -4.56 -5.82 6.54 10.93 9.88 -11.92 7.47 11.35 -10.86 -6.21 -4.83 1.8 7.61 8.2 |
| CORJ870103 | -0.56 -0.26 -2.87 -4.31 1.78 -2.31 -2.35 -1.35 0.81 3.83 4.09 -4.08 3.11 3.67 -3.22 -1.85 -1.97 -0.11 2.17 3.31 |
| CORJ870104 | 1.37 1.33 6.29 8.93 -4.47 3.88 4.04 3.39 -1.65 -7.92 -8.68 7.7 -7.13 -7.96 6.25 4.08 4.02 0.79 -4.73 -6.94 |
| CORJ870105 | -0.02 0.44 0.63 0.72 -0.96 0.56 0.74 0.38 0.0 -1.89 -2.29 1.01 -1.36 -2.22 0.47 0.55 0.25 -1.28 -0.88 -1.34 |
| CORJ870106 | 0.0 0.07 0.1 0.12 -0.16 0.09 0.12 0.06 0.0 -0.31 -0.37 0.17 -0.22 -0.36 0.08 0.09 0.04 -0.21 -0.14 -0.22 |
| CORJ870107 | -0.03 0.09 0.13 0.17 -0.36 0.13 0.23 0.09 -0.04 -0.33 -0.38 0.32 -0.3 -0.34 0.2 0.1 0.01 -0.24 -0.23 -0.29 |
| CORJ870108 | -0.04 0.07 0.13 0.19 -0.38 0.14 0.23 0.09 -0.04 -0.34 -0.37 0.33 -0.3 -0.38 0.19 0.12 0.03 -0.33 -0.29 -0.29 |
| MIYS990101 | -1.6 12.3 4.8 9.2 -2.0 4.1 8.2 -1.0 3.0 -3.1  -2.8 8.8 -3.4 -3.7 0.2 -0.6 -1.2 -1.9 0.7 -2.6 |
| MIYS990102 | -0.21 2.11 0.96 1.36 -6.04 1.52 2.3 0.0 -1.23 -4.81  -4.68 3.88 -3.66 -4.65 0.75 1.74 0.78 -3.32 -1.01 -3.5 |

Table 4. Performance (AUC) of individual structure from CluSMOTE DT and available peers from the independent test set from Kringelum et al., 2012

| No | PDB id | **Epitope** | **Antigen** | ***DiscoTope-­‐*** | ***ElliPro*** | ***Epitopia*** | ***EPCES*** | ***PEPITO*** | ***DiscoTope-­‐*** | ***CluSMOTEDT*** |
| --- | --- | --- | --- | --- | --- | --- | --- | --- | --- | --- |
|  |  | **Size** | **size** | ***1.2*** |  |  |  |  | ***2.0*** |  |
| 1 | 1YNT.F | 19 | 252 | 0,491 | 0,710 | 0,577 | 0,737 | 0,507 | 0,410 | *0,779* |
| 2 | 2NYY.A | 23 | 1268 | 0,617 | 0,769 | 0,833 | 0,700 | 0,735 | 0,795 | *0,807* |
| 3 | 2NZ9.A | 25 | 1268 | 0,597 | 0,752 | 0,796 | 0,716 | 0,728 | 0,789 | *0,791* |
| 4 | 3I50.E | 12 | 273 | 0,577 | 0,945 | 0,473 | 0,385 | 0,834 | 0,962 | *0,877* |
| 5 | 1Z3G.A | 19 | 173 | 0,689 | 0,759 | 0,744 | 0,787 | 0,761 | 0,813 | *0,813* |
| 6 | 2R56.A, B | 21 | 159 | 0,583 | 0,666 | 0,659 | 0,629 | 0,645 | 0,661 | 0,772 |
| 7 | 3BSZ.E,F | 11 | 174 | 0,658 | 0,705 | 0,587 | 0,498 | 0,633 | 0,628 | 0,824 |
| 8 | 2I9L.I | 14 | 173 | 0,535 | 0,785 | 0,661 | 0,445 | 0,652 | 0,770 | 0,783 |
| 9 | 3G04.C | 23 | 228 | 0,416 | 0,411 | 0,507 | 0,750 | 0,445 | 0,438 | 0,841 |
| 10 | 3KJ4.A | 19 | 283 | 0,514 | 0,722 | 0,661 | 0,544 | 0,467 | 0,366 | 0,730 |
| 11 | 3O0R.B, C | 9 | 449 | 0,885 | 0,868 | 0,834 | 0,723 | 0,900 | 0,891 | 0,888 |
| 12 | 2Q8A.A | 21 | 316 | 0,811 | 0,776 | 0,673 | 0,761 | 0,790 | 0,779 | *0,862* |
| 13 | 2Q8B.A | 21 | 292 | 0,886 | 0,822 | 0,630 | 0,749 | 0,857 | 0,867 | *0,894* |
| 14 | 3GI8.C | 16 | 435 | 0,920 | 0,761 | 0,899 | 0,645 | 0,912 | 0,945 | *0,803* |
| 15 | 3GI9.C | 16 | 437 | 0,921 | 0,780 | 0,918 | 0,569 | 0,919 | 0,949 | *0,811* |
| 16 | 3NCY.C, D | 12 | 420 | 0,928 | 0,664 | 0,960 | 0,920 | 0,907 | 0,934 | *0,943* |
| 17 | 2DD8.S | 19 | 192 | 0,634 | 0,629 | 0,682 | 0,730 | 0,679 | 0,720 | 0,738 |
| 18 | 2GHW.A | 24 | 191 | 0,729 | 0,755 | 0,734 | 0,750 | 0,836 | 0,901 | 0,828 |
| 19 | 3BGF.S | 15 | 179 | 0,611 | 0,636 | 0,616 | 0,763 | 0,649 | 0,666 | 0,791 |
| 20 | 2XQY.A | 19 | 464 | 0,691 | 0,541 | 0,605 | 0,710 | 0,674 | 0,667 | *0,859* |
| 21 | 2NR6.A | 18 | 330 | 0,725 | 0,783 | 0,877 | 0,869 | 0,710 | 0,695 | 0,810 |
| No | PDB id | **Epitope** | **Antigen** | ***DiscoTope-­‐*** | ***ElliPro*** | ***Epitopia*** | ***EPCES*** | ***PEPITO*** | ***DiscoTope-­‐*** | ***CluSMOTEDT*** |
|  |  | **size** | **size** | ***1.2*** |  |  |  |  | ***2*** |  |
| 22 | 3LIZ.A | 18 | 329 | 0,647 | 0,809 | 0,538 | 0,499 | 0,693 | 0,712 | 0,765 |
| 23 | 2ZJS.Y | 11 | 415 | 0,912 | 0,946 | 0,946 | 0,732 | 0,938 | 0,959 | 0,853 |
| 24 | 2UZI.R | 18 | 166 | 0,724 | 0,685 | 0,760 | 0,643 | 0,701 | 0,641 | *0,839* |
| 25 | 2VH5.R | 17 | 166 | 0,743 | 0,696 | 0,835 | 0,644 | 0,717 | 0,668 | *0,847* |
| 26 | 3R1G.B | 19 | 381 | 0,721 | 0,913 | 0,634 | 0,768 | 0,754 | 0,726 | *0,868* |
| 27 | 2ZCH.P | 16 | 237 | 0,837 | 0,674 | 0,459 | 0,698 | 0,794 | 0,758 | 0,813 |
| 28 | 2ZCK.P | 20 | 237 | 0,822 | 0,677 | 0,473 | 0,690 | 0,800 | 0,748 | 0,777 |
| 29 | 2ZCL.P | 18 | 237 | 0,833 | 0,671 | 0,451 | 0,696 | 0,801 | 0,765 | 0,820 |
| 30 | 3BN9.B | 24 | 241 | 0,614 | 0,585 | 0,641 | 0,742 | 0,599 | 0,547 | 0,752 |
| 31 | 3L95.Y | 22 | 218 | 0,524 | 0,568 | 0,445 | 0,544 | 0,501 | 0,443 | *0,796* |
| 32 | 3MXW.A | 22 | 155 | 0,649 | 0,524 | 0,484 | 0,635 | 0,642 | 0,639 | *0,826* |
| 33 | 2R4R.A | 11 | 216 | 0,964 | 0,885 | 0,906 | 0,973 | 0,975 | 0,978 | *0,942* |
| 34 | 2R4S.A | 12 | 216 | 0,967 | 0,890 | 0,895 | 0,977 | 0,978 | 0,981 | *0,948* |
| 35 | 3KJ6.A | 11 | 222 | 0,971 | 0,900 | 0,922 | 0,962 | 0,976 | 0,979 | *0,913* |
| 36 | 2OZ4.A | 18 | 265 | 0,790 | 0,401 | 0,275 | 0,746 | 0,786 | 0,789 | 0,850 |
| 37 | 2FD6.U | 13 | 249 | 0,752 | 0,682 | 0,598 | 0,745 | 0,790 | 0,706 | 0,787 |
| 38 | 3PGF.A | 24 | 358 | 0,506 | 0,367 | 0,560 | 0,539 | 0,439 | 0,337 | 0,794 |
| 39 | 2J88.A | 10 | 319 | 0,961 | 0,992 | 0,487 | 0,579 | 0,989 | 0,989 | *0,917* |
|  | average |  |  | 0,727 | 0,721 | 0,673 | 0,697 | 0,746 | 0,744 | 0,830 |

Note: Test results on the comparable method are taken from supplements in Kringelum et al., 2012

Table 5. Performance (AUC) of individual structure from CluSMOTE DT and available peers from the independent test set from Chou et al., 2019

| **No** | PDB id | SEPPA  3.0 | SEPPA  2.0 | Pepito | Epitopia | Discotope  2.0 | CBTOPE | Bepipred  2.0 | CluSMOTE DT |
| --- | --- | --- | --- | --- | --- | --- | --- | --- | --- |
| **1** | 3JCX_A | 0,681 | 0,551 | 0,764 | 0,763 | 0,725 | 0,532 | 0,546 | 0,706 |
| **2** | 5B3J_A | 0,812 | 0,491 | 0,783 | 0,774 | 0,796 | 0,476 | 0,701 | 0,801 |
| **3** | 5B3J_D | 0,788 | 0,523 | 0,645 | 0,777 | 0,654 | 0,479 | 0,623 | 0,841 |
| **4** | 5B8C_C | 0,664 | 0,651 | 0,659 | 0,549 | 0,723 | 0,543 | 0,628 | 0,799 |
| **5** | 5FUU_C | 0,704 | 0,582 | 0,459 | 0,687 | 0,584 | 0,509 | 0,660 | 0,858 |
| **6** | 5FUU_F | 0,654 | 0,565 | 0,592 | 0,697 | 0,716 | 0,500 | 0,836 | 0,551 |
| **7** | 5FV1_V | 0,554 | 0,498 | 0,596 | 0,683 | 0,704 | 0,722 | 0,636 | 0,769 |
| **8** | 5FV2_X | 0,643 | 0,509 | 0,605 | 0,731 | 0,709 | 0,635 | 0,595 | 0,777 |
| **9** | 5FYL_G | 0,870 | 0,702 | 0,768 | 0,845 | 0,772 | 0,493 | 0,618 | 0,708 |
| **10** | 5FYL_B | 0,606 | 0,471 | 0,656 | 0,759 | 0,610 | 0,488 | 0,736 | 0,716 |
| **11** | 5GGR_Z | 0,984 | 0,968 | 0,803 | 0,453 | 0,895 | 0,593 | 0,815 | 0,894 |
| **12** | 5GGS_Z | 0,696 | 0,718 | 0,639 | 0,542 | 0,702 | 0,524 | 0,573 | 0,832 |
| **13** | 5GGV_Y | 0,538 | 0,629 | 0,641 | 0,535 | 0,641 | 0,484 | 0,627 | 0,814 |
| **14** | 5GJS_A | 0,646 | 0,573 | 0,788 | 0,647 | 0,840 | 0,528 | 0,541 | 0,893 |
| **15** | 5GJS_B | 0,901 | 0,647 | 0,572 | 0,534 | 0,522 | 0,442 | 0,549 | 0,739 |
| **16** | 5GJT_A | 0,685 | 0,597 | 0,700 | 0,630 | 0,738 | 0,563 | 0,622 | 0,896 |
| **17** | 5GJT_B | 0,817 | 0,624 | 0,563 | 0,571 | 0,508 | 0,439 | 0,525 | 0,725 |
| **18** | 5GRJ_A | 0,658 | 0,593 | 0,465 | 0,521 | 0,546 | 0,546 | 0,570 | 0,786 |
| **19** | 5GS0_A | 0,816 | 0,385 | 0,673 | 0,713 | 0,576 | 0,647 | 0,675 | 0,735 |
| **20** | 5GZN_A | 0,530 | 0,618 | 0,766 | 0,624 | 0,740 | 0,560 | 0,503 | 0,760 |
| **21** | 5H37_A | 0,920 | 0,702 | 0,605 | 0,701 | 0,728 | 0,703 | 0,658 | 0,739 |
| **22** | 5HDQ_A | 0,602 | 0,864 | 0,484 | 0,858 | 0,554 | 0,430 | 0,521 | 0,809 |
| **23** | 5HHX_A | 0,760 | 0,491 | 0,712 | 0,738 | 0,793 | 0,630 | 0,482 | 0,803 |
| **24** | 5HI3_B | 0,923 | 0,641 | 0,703 | 0,689 | 0,788 | 0,675 | 0,467 | 0,671 |
| **25** | 5HI4_A | 0,657 | 0,543 | 0,664 | 0,644 | 0,706 | 0,679 | 0,480 | 0,636 |
| **26** | 5HI5_A | 0,910 | 0,869 | 0,883 | 0,448 | 0,833 | 0,259 | 0,905 | 0,686 |
| **27** | 5HJ3_D | 0,527 | 0,742 | 0,599 | 0,713 | 0,834 | 0,954 | 0,765 | 0,516 |
| **28** | 5I6X_A | 0,603 | 0,615 | 0,925 | 0,824 | 0,932 | 0,684 | 0,846 | 0,963 |
| **29** | 5I6Z_A | 0,754 | 0,540 | 0,913 | 0,825 | 0,912 | 0,752 | 0,841 | 0,963 |
| **30** | 5I74_A | 0,724 | 0,669 | 0,921 | 0,829 | 0,930 | 0,728 | 0,843 | 0,961 |
| **31** | 5I8H_C | 0,895 | 0,586 | 0,767 | 0,738 | 0,769 | 0,508 | 0,636 | 0,747 |
| **32** | 5I8H_B | 0,612 | 0,798 | 0,465 | 0,782 | 0,618 | 0,805 | 0,715 | 0,726 |
| **33** | 5I9Q_G | 0,834 | 0,682 | 0,776 | 0,585 | 0,798 | 0,512 | 0,723 | 0,777 |
| **34** | 5IES_C | 0,857 | 0,861 | 0,878 | 0,667 | 0,885 | 0,614 | 0,766 | 0,777 |
| **35** | 5IF0_G | 0,936 | 0,841 | 0,885 | 0,645 | 0,895 | 0,645 | 0,773 | 0,777 |
| **36** | 5IGX_G | 0,866 | 0,780 | 0,821 | 0,690 | 0,863 | 0,513 | 0,690 | 0,777 |
| **37** | 5J3H_E | 0,939 | 0,530 | 0,609 | 0,741 | 0,634 | 0,810 | 0,639 | 0,844 |
| **38** | 5J56_A | 0,865 | 0,860 | 0,542 | 0,732 | 0,505 | 0,366 | 0,566 | 0,811 |
| **39** | 5J57_A | 0,677 | 0,781 | 0,747 | 0,829 | 0,734 | 0,601 | 0,689 | 0,805 |
| **40** | 5JQ6_A | 0,504 | 0,690 | 0,780 | 0,695 | 0,755 | 0,546 | 0,552 | 0,820 |
| **41** | 5JS9_C | 0,863 | 0,504 | 0,721 | 0,831 | 0,670 | 0,374 | 0,641 | 0,753 |
| **42** | 5JS9_D | 0,859 | 0,466 | 0,857 | 0,843 | 0,773 | 0,444 | 0,520 | 0,741 |
| **43** | 5JSA_D | 0,961 | 0,586 | 0,871 | 0,838 | 0,801 | 0,484 | 0,560 | 0,727 |
| **44** | 5JW3_A | 0,904 | 0,838 | 0,737 | 0,640 | 0,757 | 0,433 | 0,489 | 0,872 |
| **45** | 5JW3_B | 0,702 | 0,583 | 0,525 | 0,579 | 0,615 | 0,450 | 0,486 | 0,709 |
| **46** | 5JW4_A | 0,803 | 0,562 | 0,729 | 0,793 | 0,798 | 0,633 | 0,534 | 0,839 |
| **47** | 5JXE_B | 0,546 | 0,722 | 0,686 | 0,577 | 0,772 | 0,498 | 0,592 | 0,817 |
| **48** | 5JYL_A | 0,839 | 0,853 | 0,750 | 0,610 | 0,725 | 0,447 | 0,745 | 0,777 |
| **49** | 5JYM_A | 0,542 | 0,724 | 0,613 | 0,592 | 0,599 | 0,581 | 0,535 | 0,765 |
| **50** | 5JZ7_B | 0,653 | 0,678 | 0,527 | 0,579 | 0,667 | 0,454 | 0,551 | 0,753 |
| **51** | 5K59_A | 0,733 | 0,735 | 0,802 | 0,764 | 0,731 | 0,463 | 0,659 | 0,787 |
| **52** | 5K9K_I | 0,603 | 0,659 | 0,565 | 0,674 | 0,616 | 0,390 | 0,582 | 0,727 |
| **53** | 5K9O_I | 0,627 | 0,506 | 0,565 | 0,477 | 0,588 | 0,493 | 0,592 | 0,723 |
| **54** | 5K9Q_F | 0,883 | 0,569 | 0,646 | 0,716 | 0,552 | 0,296 | 0,642 | 0,673 |
| **55** | 5KAN_C | 0,539 | 0,569 | 0,618 | 0,789 | 0,591 | 0,634 | 0,716 | 0,646 |
| **56** | 5KAN_D | 0,922 | 0,539 | 0,637 | 0,615 | 0,532 | 0,283 | 0,662 | 0,548 |
| **57** | 5KAQ_B | 0,875 | 0,466 | 0,627 | 0,561 | 0,654 | 0,313 | 0,690 | 0,666 |
| **58** | 5KEL_B | 0,695 | 0,464 | 0,567 | 0,758 | 0,757 | 0,951 | 0,779 | 0,574 |
| **59** | 5KEL_A | 0,570 | 0,546 | 0,760 | 0,733 | 0,823 | 0,464 | 0,611 | 0,792 |
| **60** | 5KEN_E | 0,661 | 0,567 | 0,723 | 0,743 | 0,774 | 0,502 | 0,521 | 0,638 |
| **61** | 5KEN_B | 0,650 | 0,441 | 0,645 | 0,748 | 0,807 | 0,910 | 0,726 | 0,569 |
| **62** | 5KJR_G | 0,570 | 0,559 | 0,646 | 0,571 | 0,641 | 0,390 | 0,659 | 0,731 |
| **63** | 5KOV_A | 0,628 | 0,783 | 0,685 | 0,770 | 0,679 | 0,548 | 0,603 | 0,799 |
| **64** | 5KTE_A | 0,735 | 0,725 | 0,733 | 0,832 | 0,758 | 0,711 | 0,805 | 0,796 |
| **65** | 5KW9_A | 0,684 | 0,624 | 0,847 | 0,683 | 0,897 | 0,588 | 0,618 | 0,907 |
| **66** | 5KZC_A | 0,709 | 0,783 | 0,881 | 0,622 | 0,885 | 0,579 | 0,749 | 0,771 |
| **67** | 5L0Q_A | 0,682 | 0,445 | 0,663 | 0,574 | 0,602 | 0,410 | 0,557 | 0,774 |
| **68** | 5L6Y_C | 0,713 | 0,694 | 0,775 | 0,464 | 0,785 | 0,547 | 0,540 | 0,853 |
| **69** | 5M94_A | 0,897 | 0,751 | 0,615 | 0,788 | 0,593 | 0,549 | 0,800 | 0,741 |
| **70** | 5MVZ_U | 0,727 | 0,609 | 0,594 | 0,562 | 0,542 | 0,769 | 0,556 | 0,853 |
| **71** | 5SV3_B | 0,841 | 0,815 | 0,761 | 0,597 | 0,715 | 0,508 | 0,652 | 0,819 |
| **72** | 5SX4_N | 0,845 | 0,728 | 0,586 | 0,595 | 0,604 | 0,915 | 0,655 | 0,803 |
| **73** | 5SX5_N | 0,868 | 0,618 | 0,628 | 0,672 | 0,630 | 0,914 | 0,657 | 0,818 |
| **74** | 5T33_G | 0,909 | 0,874 | 0,900 | 0,712 | 0,928 | 0,403 | 0,810 | 0,559 |
| **75** | 5T3S_G | 0,776 | 0,524 | 0,606 | 0,668 | 0,576 | 0,398 | 0,619 | 0,805 |
| **76** | 5T3Z_G | 0,615 | 0,542 | 0,771 | 0,529 | 0,730 | 0,494 | 0,591 | 0,722 |
| **77** | 5TE4_G | 0,886 | 0,662 | 0,777 | 0,562 | 0,767 | 0,377 | 0,623 | 0,786 |
| **78** | 5TE6_G | 0,951 | 0,863 | 0,787 | 0,543 | 0,801 | 0,568 | 0,622 | 0,534 |
| **79** | 5TE7_G | 0,935 | 0,763 | 0,772 | 0,566 | 0,764 | 0,390 | 0,607 | 0,545 |
| **80** | 5TH9_A | 0,716 | 0,889 | 0,777 | 0,799 | 0,761 | 0,349 | 0,590 | 0,834 |
| **81** | 5THR_C | 0,634 | 0,671 | 0,783 | 0,683 | 0,722 | 0,474 | 0,515 | 0,627 |
| **82** | 5THR_D | 0,908 | 0,849 | 0,502 | 0,622 | 0,446 | 0,708 | 0,525 | 0,696 |
| **83** | 5TOJ_A | 0,647 | 0,590 | 0,459 | 0,605 | 0,536 | 0,563 | 0,647 | 0,772 |
| **84** | 5TOK_B | 0,561 | 0,600 | 0,575 | 0,647 | 0,500 | 0,579 | 0,641 | 0,779 |
| **85** | 5TPN_A | 0,616 | 0,475 | 0,525 | 0,611 | 0,507 | 0,464 | 0,719 | 0,852 |
| **86** | 5TPW_A | 0,835 | 0,459 | 0,586 | 0,653 | 0,569 | 0,495 | 0,642 | 0,855 |
| **87** | 5TQ0_A | 0,854 | 0,546 | 0,543 | 0,615 | 0,548 | 0,489 | 0,627 | 0,827 |
| **88** | 5TQ2_A | 0,812 | 0,510 | 0,539 | 0,541 | 0,532 | 0,501 | 0,694 | 0,780 |
| **89** | 5TQQ_A | 0,602 | 0,774 | 0,815 | 0,735 | 0,833 | 0,519 | 0,765 | 0,831 |
| **90** | 5TR1_A | 0,567 | 0,710 | 0,833 | 0,750 | 0,853 | 0,520 | 0,790 | 0,884 |
| **91** | 5TZ2_C | 0,785 | 0,571 | 0,613 | 0,728 | 0,540 | 0,447 | 0,507 | 0,875 |
| **92** | 5TZT_C | 0,689 | 0,728 | 0,514 | 0,727 | 0,473 | 0,458 | 0,460 | 0,812 |
| **93** | 5TZU_C | 0,479 | 0,676 | 0,570 | 0,561 | 0,514 | 0,409 | 0,522 | 0,868 |
| **94** | 5UGY_A | 0,899 | 0,610 | 0,771 | 0,642 | 0,849 | 0,492 | 0,559 | 0,525 |
| **95** | 5UQY_E | 0,571 | 0,525 | 0,600 | 0,559 | 0,434 | 0,572 | 0,662 | 0,688 |
| **96** | 5WT9_G | 0,948 | 0,980 | 0,884 | 0,473 | 0,948 | 0,610 | 0,839 | 0,891 |
|  | mean | 0,739 | 0,645 | 0,684 | 0,672 | 0,699 | 0,545 | 0,638 | 0,766 |

Note: Test results on the comparable method are taken from supplements in Chou et al., 2019
